# Effects of externally-applied, non-pharmacological Interventions on short- and long-term symptoms and inflammatory cytokine levels in patients with knee osteoarthritis: a systematic review and network meta-analysis

**DOI:** 10.3389/fimmu.2023.1309751

**Published:** 2023-12-14

**Authors:** Zhen Wang, Hui Xu, Zheng Wang, Hang Zhou, Jieyao Diao, Lijuan Zhang, Yu Wang, Miaoxiu Li, Yunfeng Zhou

**Affiliations:** ^1^ College of Acupuncture and Massage, Henan University of Chinese Medicine, Zhengzhou, China; ^2^ Tuina Department, The Third Affiliated Hospital of Henan University of Chinese Medicine, Zhengzhou, China; ^3^ Rehabilitation Department, Jiaozuo Coal Industry (Group) Co. Ltd., Central Hospital, Jiaozuo, China; ^4^ College of Computer Science, Xidian University, Xian, China; ^5^ College of Acupuncture and Massage, Shanghai University of Chinese Medicine, Shanghai, China

**Keywords:** knee osteoarthritis, externally-applied, non-pharmacological interventions, short- and long-term, efficacy, inflammatory cytokine, network meta-analysis

## Abstract

**Background:**

With the continuous development of clinical medicine, an increasing number of non-pharmacological interventions have been applied for the treatment of knee osteoarthritis (KOA), with the results of several recent randomized controlled trials (RCTs) showing that a variety of externally-applied, non-pharmacological interventions (EANPI) can improve symptoms and inflammation in patients with KOA. However, the relative benefits and disadvantages of non-drug therapies remain uncertain, and an optimal treatment strategy has not yet been determined.

**Objective:**

This study applied network meta-analysis (NMA) to compare and rank the effectiveness of EANPI on the short- and long-term clinical symptoms and inflammatory cytokine levels in patients with KOA.

**Methods:**

Two independent researchers searched online databases and performed manual retrieval of related citations to identify RCTs that met the selection criteria for the network meta-analysis. These researchers retrieved studies indexed from database inception to August 2023 and performed data extraction and assessment of the risk of bias.

**Results:**

The analysis included 80 RCTs involving 8440 participants and nine externally-applied, non-pharmacological therapies, namely extracorporeal shock wave, radiofrequency, acupotomy, laser therapy, Tuina therapy, kinesio taping, electroacupuncture, platelet-rich plasma injection, and ozone therapy. The treatment courses ranged from 1 to 12 weeks, with follow-up periods ranging from 4 to 24 weeks. The results of the NMA indicated that each non-drug therapy was superior to sham intervention in improving all outcome indicators. Except for the visual analog scale (VAS) and Western Ontario MacMaster (WOMAC) pain outcomes, all non-drug therapies had better efficacy than pharmacological treatments. For short-term VAS and tumor necrosis factor-alpha (TNF-α), extracorporeal shock wave performed better than other therapies (90.2% and 85.2% respectively). Radiofrequency therapy may be the most promising method to reduce long-term VAS, short- and long-term WOMAC pain, and interleukin (IL)-1β level (84.8%, 97.8%, 90.1%, 94.8% respectively). Tuina therapy may be a significant choice for short- and long-term outcomes of WOMAC function and range of motion (ROM).

**Conclusions:**

The results of the comprehensive comparison of the outcome indicators in 9 different EANPI indicated that radiofrequency and Tuina therapy were more effective and consistently ranked high in improving clinical symptoms in the short and long term. Radiofrequency is effective at relieving pain, and Tuina therapy can be given priority for treatment when hypofunction is the main symptom. EANPI to improve pain symptoms may be related to the regulation of inflammatory cytokine levels, which may be a potential mechanism of action.

**Systematic review registration:**

https://www.crd.york.ac.uk/prospero/display_record.php?, identifier CRD42023464177.

## 1 Introduction

Knee osteoarthritis (KOA) is the most common type of osteoarthritis and mainly manifests as knee pain, swelling, and unfavorable flexion and extension (1, 2). Globally, KOA is the 11th leading cause of disability, affecting approximately 3.8% of the population (3). Owing to the increase in work pressures and acceleration in the pace of life, the annual incidence of KOA has increased rapidly (4). The pathogenesis of KOA is complex and involves several inflammatory cytokines. Inflammatory factors are involved in processes such as chondrocyte damage, extracellular matrix degradation, and bone redundancy, which play important roles in KOA development (5, 6). Drug therapy can prevent or reduce joint damage and maintain normal joint function (7, 8). Although various types of drug therapies have been used for the treatment of KOA, including non-steroidal anti-inflammatory drugs (NSAIDs), sodium hyaluronate injection, and topical voltaline, the shortcomings include adverse reactions, poor long-term efficacy, and easily reached treatment bottleneck (9, 10). Therefore, the optimization of KOA treatment strategies is a major concern for clinicians.

Concerns regarding the safety and bottlenecking of drug treatments have increased the focus on non-drug therapies. Non-drug treatments for KOA have the advantages of significantly higher efficacy, lasting effects, and few adverse reactions, and have become a hot research topic in recent years (11, 12). Several guidelines and consensuses (13–15) list non-drug therapies as recommended interventions for the clinical treatment of KOA. However, the various types of non-pharmacological interventions include radiofrequency, extracorporeal shock wave, kinesio taping, and massage, and a direct comparison of the curative effects of different non-drug therapies is lacking. Therefore, the choice of non-drug therapy for KOA remains controversial.

While several traditional meta-analyses (16–19) have demonstrated the advantages of non-drug treatment of KOA, these analyses have focused on the comparison of a single non-drug therapy with drugs or another non-drug therapy and have not compared multiple non-pharmacological interventions simultaneously. As the number of alternative treatment options increases, comparative effectiveness studies will be necessary. To date, no meta-analysis has comprehensively compared and evaluated the efficacy of multiple types of non-drug therapies. Thus, the intervention measures with the best effects are unknown. In addition, most systematic reviews have focused only on short-term changes in clinical symptom indicators and have failed to explore long-term outcomes and changes in inflammatory cytokine levels in patients with KOA treated with non-pharmacological interventions. Therefore, a network meta-analysis (NMA) was performed to simultaneously analyze both direct and indirect evidence from different studies, estimate the relative effectiveness of all interventions, and rank the order of interventions (20, 21). This study systematically evaluated the effects of non-pharmacological therapies on short- and long-term outcomes and inflammatory cytokines in patients with KOA to provide evidence for choosing the best plans for the clinical treatment of patients with KOA.

## 2 Methods

### 2.1 Study protocol and registration

The NMA and systematic review were conducted strictly in accordance with the Preferred Reporting Items for Systematic Reviews and Meta-Analyses (PRISMA-NMA) guidelines (22) (see **Supplementary Table S1**). The study protocol is registered in the International Prospective Register of Systematic Reviews (PROSPERO) (CRD42023464177).

### 2.2 Inclusion criteria

#### 2.2.1 Research type

Only randomized controlled trials (RCTs) were included and were not restricted to any language.

#### 2.2.2 Research objects

All studies met the recognized diagnostic criteria for KOA, regardless of age, sex, or race.

#### 2.2.3 Interventions

The patients in the treatment group received only externally-applied, non-pharmacological interventions. Patients in the control group were treated with a sham intervention, conventional medicine, or any non-pharmacological intervention in the treatment group. The inclusion of intervention drugs in the control group was based on accepted guidelines or consensus (23, 24). Conventional medicines are divided into oral and non-oral drugs (NOD); oral drugs are only included as NSAIDs.

#### 2.2.4 Outcome indicators

(1) Pain: Visual analog scale (VAS), Western Ontario MacMaster (WOMAC) pain score (2); Function: WOMAC function score, Joint range of motion (ROM) (3); Inflammatory cytokine: Interleukin-1β (IL-1β), tumor necrosis factor (TNF-α). All outcome measures were analyzed after treatment to determine short-term efficacy. In addition, the long-term effects of non-pharmacological therapies on pain and functional indicators were analyzed during follow-up.

### 2.3 Exclusion criteria

The exclusion criteria were (1) patients with other inflammatory diseases (2), repeated publications, (3) more than one therapy, (4) no reference or homemade diagnostic criteria, (5) unavailability of full texts and outcomes, and (6) serious complications.

### 2.4 Literature search strategy

The Cochrane Library, Embase, PubMed, Web of Science, Chinese Biomedical Database (CBM), VIP, Chinese National Knowledge Infrastructure (CNKI), and Wanfang databases were searched for relevant studies. Grey literature was manually searched, and the reference catalogs included in each study and related systematic reviews were consulted. The retrieval strategy used a combination of subject headings and free words. The databases were searched from their inception to August 20, 2023. An example of the PubMed search strategy is shown in **Supplementary Figure S1**.

### 2.5 Literature screening and data extraction

Two researchers (WY and ZL) independently screened the studies based on the inclusion criteria. EndNote software was used to check for duplicate studies. The investigators screened the titles and abstracts of each study and excluded studies that did not meet the inclusion criteria. Subsequently, the investigators read the full texts of the remaining studies to decide whether to include them. Disagreements were resolved through consultations with a third party (LX). Two reviewers (LM and WZ) separately extracted the data from each eligible RCT using a standardized form. The extracted data included the study characteristics (author, country, and publication date), patient characteristics (sample size, disease duration, sex, and age), research site, methodology, intervention measures, treatment course, follow-up, and outcome indicators.

### 2.6 Risk of bias assessment

The risk of bias in the included studies was evaluated by two separate researchers (WY and ZH) using the RCT Bias Risk Assessment Tool of the Cochrane System Review Manual, version 6.1.0 (25). A third investigator (XH) assisted in resolving differences in assessments between the two researchers. The evaluation items included random sequence generation, allocation concealment, blinding of participants and personnel, blinding of outcome assessments, incomplete data, selective reporting, and other biases. Finally, the included studies were categorized as having low, high, or unclear risks of bias.

### 2.7 Statistical analysis

All outcome indicators were analyzed using random- or fixed-effects models, based on the level of heterogeneity. The P-values of the chi-square test and the I^2^ index in the heterogeneity test were used to indicate the level of statistical heterogeneity (26). When the level of heterogeneity was low, the data were analyzed using the fixed-effects model (P ≥0.1 and I^2^ <50%); otherwise, the random-effects model (P <0.1 or I^2^ <50%) was used (27). As the indicators to be analyzed were all continuous variables, we chose the standardized mean difference (SMD) as the effect scale. All results are presented as 95% confidence intervals (CIs).

Based on the Bayesian model, Stata software (version 16.0, StataCorp LLC, College Station, TX, USA) was used to perform the network meta-analysis. The data were preprocessed using the network group command, and an evidence network diagram was drawn for each indicator. The curative effects of the indicators were sorted to obtain the surface under the cumulative ranking curve (SUCRA), and the probability sorting was plotted. The dots in the evidence network diagram represent an intervention; the larger the area, the greater the number of patients receiving the intervention. The line connecting the two dots indicates a direct comparison between the two interventions, while the thickness of the line represents the number of included studies (28, 29). SUCRA was expressed as a percentage, with a larger percentage indicating that the intervention has the highest probability of becoming the preferred option, and a value of zero indicating that the intervention may be completely ineffective (30). When a closed loop existed, the node-splitting method was used to check for inconsistencies. When >10 studies assessed the outcome indicator, funnel plots were drawn to determine the possibility of a small sample effect (31). To test the robustness of the findings, some factors that might have influenced the level of precision of the main outcome were removed and a sensitivity analysis was performed. Subgroup analyses were performed based on different treatment courses and follow-up cycles. Finally, the quality of the literature was evaluated using Review Manager 5.4 software.

## 3 Results

### 3.1 Literature screen results

**Figure 1:**
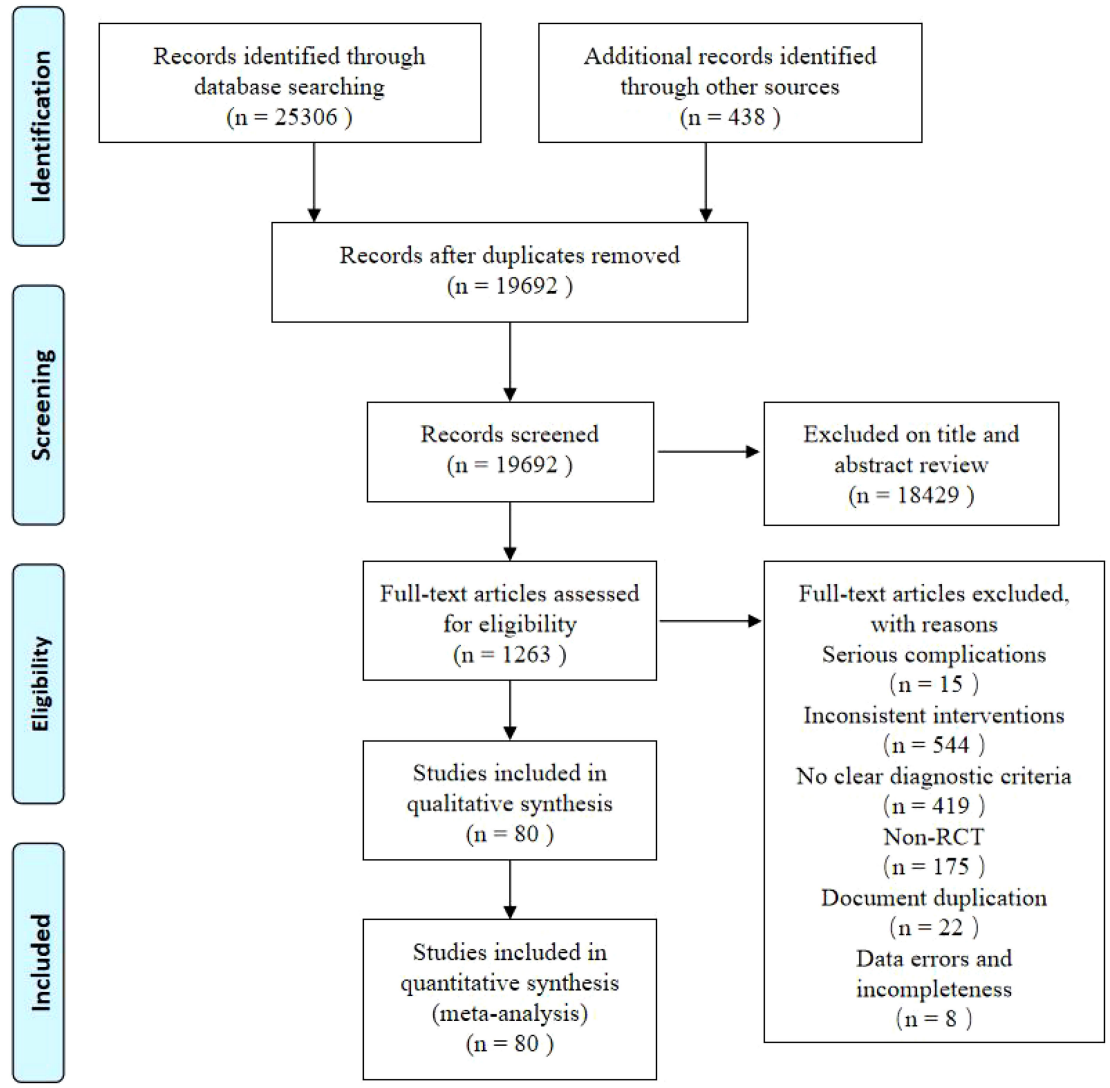
Literature screening process.

Of the 25,744 potentially relevant references identified (25,306 from each database and 438 from supplementary searches), 19692 articles were left after removing duplicates. A total of 1263 studies were subjected to full-text screening after title and abstract screening. Finally, the NMA included 80 RCTs (32–111). The screening flowchart is shown in **Figure 1**.

### 3.2 Characteristics of the included studies

The basic characteristics of the included studies (n=80) are presented in **Table 1**. A total of 8440 subjects were included in this study, with 4242 in the treatment group and 4198 in the control group, respectively. Thirteen studies (43, 50, 64, 66, 73, 87, 91, 99, 102–104, 108, 111) were multi-center trials and the rest were single-center studies. The sample sizes of the included studies ranged from 39 to 615. The intervention durations ranged from 1 to 12 weeks. Forty-five studies (39, 41, 43–46, 49–55, 58, 60, 62, 64–66, 68, 69, 73, 74, 78, 79, 82, 85, 86, 88–96, 100, 101, 104, 107–111) reported follow-up durations ranging from 4 to 24 weeks. The nine non-drug therapies included extracorporeal shock waves (ESW), radiofrequency (RF), acupotomy (AT), laser therapy (LT), Tuina therapy (TT), kinesio taping (KT), electroacupuncture (EA), platelet-rich plasma injection (PRP), and ozone therapy (OT). The descriptions of each non-pharmacological intervention are presented in **Table 2**.

**Table 1:** Characteristics of the included studies.

Included Studies	Mean age/years		Sample size(M/F)		Mean disease duration/year		Interventions		Duration /week	Follow up /week	Outcome Measures
	T	C	T	C	T	C	T	C			
Dong B 2022 (32)	68.02 ± 6.36	67.74 ± 6.48	22/15	20/17	2.54 ± 0.68yr	2.15 ± 0.78yr	ESW	NOD	12	–	①⑤⑥
Liu WF 2019 (33)	40.38 ± 3.29	40.19 ± 3.58	18/15	20/13	43.36 ± 2.57mon	43.12 ± 2.89mon	ESW	NSAIDs	4	–	①⑤⑥
Wu YL 2022 (34)	53.87 ± 5.93	54.06 ± 6.13	14/32	17/29	3.03 ± 0.98yr	2.98 ± 1.02yr	ESW	NOD	5	–	①⑤
Zheng YL 2021 (35)	62.3 ± 1.2	62.2 ± 1.4	23/24	22/25	23.1 ± 2.4mon	23.2 ± 2.6mon	ESW	NSAIDs	4	–	①⑥
Su WZ 2019 (36)	47~68	45~66	20/40	21/39	25~45mon	24~47mon	ESW	PRP	5	–	①④
Jiang LM 2017 (37)	59.1 ± 3.1	57.3 ± 4.7	11/14	13/12	————	————	ESW	TT	4	–	②③④
Liu WT 2017 (38)	59.12 ± 5.34	58.82 ± 5.11	13/17	11/17	25.16 ± 7.13mon	24.65 ± 6.22mon	ESW	AT	1	–	②③
Ji JJ 2021 (39)	69.83 ± 5.31	70.42 ± 6.68	32/22	34/20	13.53 ± 4.42mon	15.02 ± 3.86mon	ESW	NSAIDs	12	24	①⑥
Mostafa MSEM 2022 (40)	40.12 ± 9.45	46.62 ± 8.68	20	20	17.2 ± 11.6d	18.7 ± 13.3d	ESW	LT	4	–	①
Zhao Z 2013 (41)	61.8 ± 9.8	59.9 ± 11.3	11/25	14/20	–	–	ESW	SI	4	4, 12	①②③
Uysal A 2020 (42)	60.2 ± 6.3	61.8 ± 6.0	10/42	9/43	40.2 ± 21.9mon	46.8 ± 24.0mon	ESW	SI	3	4, 12	①②③④
Zhong ZY 2019 (43)	62.5 ± 8.2	63.2 ± 7.7	11/21	12/19	34.7 ± 15.4mon	34.1 ± 14.2mon	ESW	SI	5	5, 12	①②③
Wang XC 2021 (44)	57.9 ± 4.5	59.4 ± 4.1	10/10	10/11	43.4 ± 6.9mon	43.6 ± 6.1mon	RF	NOD	8	4, 12, 24	①⑤⑥
Ma JY 2017 (45)	44~65	44~65	20	20	–	–	RF	OT	4	4	①④
Zhuo ZM 2021 (46)	57.21 ± 12.38	53.01 ± 11.92	17/28	23/22	16.21 ± 6.38mon	15.32 ± 5.47mon	RF	NOD	4	4, 12, 24	①⑥
Feng WH 2022 (47)	56.67 ± 5.14	57.31 ± 5.24	19/26	20/24	17.58 ± 5.62mon	16.41 ± 5.21mon	RF	NSAIDs	4	–	①⑥
Cai LX 2022 (48)	62.18 ± 2.37	62.29 ± 2.43	24/18	25/17	7.38 ± 1.18yr	7.24 ± 1.12yr	RF	NOD	4	–	①⑤
Elawamy A 2021 (49)	47.78 ± 6.9	48.45 ± 7.7	50/50	49/51	7.9 ± 0.48yr	8.79 ± 0.48yr	RF	PRP	4	12, 24	①
Chen AF 2020 (50)	63.3 ± 10.7	62.8 ± 9.5	52/37	40/28	–	–	RF	NOD	4	4, 12, 24	②③
Uematsu H 2021 (51)	40~85	40~85	28/9	28/5	–	–	RF	SI	4	12	①④
El-Hakeim EH 2018 (52)	62.00 ± 7.37	56.87 ± 6.53	9/21	12/18	7.60 ± 3.14yr	5.70 ± 5.10yr	RF	NSAIDs	2	12, 24	①②③
Zuo XT 2021 (53)	62.43 ± 9.04	62.13 ± 9.99	22/8	19/11	74.27 ± 20.54d	76.00 ± 20.65d	AT	NOD	3	4	①③⑥
Xiong YZ 2020 (54)	54.70 ± 9.03	52.33 ± 7.78	14/16	12/18	71.13 ± 20.33mon	66.17 ± 19.58mon	AT	NSAIDs	3	12	①②③
Meng F 2017 (55)	55.32 ± 11.41	56.46 ± 13.25	20/16	18/15	4.29 ± 1.15yr	4.77 ± 1.56yr	AT	NOD	4	12	②③⑥
Pan Q 2023 (56)	45.32 ± 5.26	45.63 ± 5.01	27/19	24/20	3.77 ± 0.39yr	3.65 ± 0.45yr	AT	NOD	4	–	②③⑤⑥
Wang C 2022 (57)	62.98 ± 6.68	64.19 ± 5.98	16/40	18/36	24.50 ± 15.25mon	25.00 ± 13.75mon	AT	NSAIDs	3	–	⑤⑥
Xu DH 2022 (58)	50~63	52~62	14/30	14/30	0.3~3.9yr	1.0~4.9yr	AT	SI	2	12	①②③④
Wang HL 2020 (59)	45~72	45~72	30	30	2~25mon	2~25mon	AT	NSAIDs	4	–	④⑤⑥
Li XP 2020 (60)	64.63 ± 6.47	63.13 ± 7.36	16/14	15/15	8.87 ± 2.89mon	8.20 ± 4.32mon	AT	EA	4	4	①②③
Jiang F 2018 (61)	62.3 ± 10.2	65.2 ± 7.9	11/13	12/12	5.3 ± 1.2yr	4.7 ± 1.4yr	LT	NOD	4	–	①④
Yurtkuran M 2007 (62)	51.83 ± 6.83	53.47 ± 7.13	1/27	1/26	61.59 ± 51.96mon	66.59 ± 57.81mon	LT	SI	2	12	①②③
Alghadir A 2013 (63)	55.2 ± 8.14	57.0 ± 7.77	10/10	12/8	9.15 ± 4.34mon	10.05 ± 3.50mon	LT	SI	4	–	①②③
Zhao L 2020 (64)	63.50 ± 7.67	63.10 ± 6.00	48/153	50/141	1~10yr	1~10yr	LT	SI	4	12, 24	①②③⑤
Helianthi DR 2016 (65)	69.00 ± 6.00	68.00 ± 5.00	12/18	5/24	–	–	LT	SI	4	–	①
Lin L 2019 (66)	64.12 ± 8.18	63.62 ± 5.60	13/84	16/77	3.00 ± 1.08yr	3.53 ± 1.13yr	LT	SI	4	4, 8, 12, 24	①②③
Shen XY 2009 (67)	60.10 ± 6.83	56.40 ± 7.41	2/18	2/18	6.05 ± 6.51yr	4.24 ± 6.65yr	LT	SI	4	–	②③
Fang G 2022 (68)	61.35 ± 9.81	62.66 ± 9.58	19/41	29/31	2.6 ± 1.2mon	2.7 ± 1.1mon	TT	NSAIDs	4	4	①②③④
Lin X 2018 (69)	60.66 ± 6.07	60.83 ± 5.86	7/21	6/21	–	–	TT	NOD	3	4, 12	②③
Li ZL 2015 (70)	64.52 ± 5.44	63.87 ± 5.32	28/33	25/32	12.93 ± 5.42yr	13.47 ± 5.67yr	TT	EA	4	–	①②③④
Li MX 2022 (71)	61.88 ± 8.76	62.45 ± 9.55	20/14	18/16	10.52 ± 2.55mon	11.68 ± 2.23mon	TT	NSAIDs	4	–	⑤
Kong LL 2020 (72)	59.43 ± 6.49	60.01 ± 8.63	15/17	17/15	7.40 ± 4.61mon	8.32 ± 4.96mon	TT	SI	4	–	①⑤⑥
Xu H 2023 (73)	63.38 ± 7.04	64.58 ± 7.56	9/43	14/38	41.81 ± 13.65mon	44.38 ± 15.58mon	TT	NSAIDs	4	4	②③
Perlman A 2019 (74)	64.3 ± 10.4	62.8 ± 10.4	16/58	9/64	–	–	TT	SI	8	8, 16, 24	①②③④
Wang XB 2020 (75)	49.9 ± 6.2	51.0 ± 6.0	9/21	12/18	6.0 ± 1.7yr	6.3 ± 2.0yr	TT	NSAIDs	2	–	①⑥
Zhao Q 2021 (76)	55.86 ± 2.14	56.25 ± 2.32	21/23	25/19	5.58 ± 0.69yr	5.62 ± 0.75yr	KT	NOD	4	–	①④
Shi BH 2020 (77)	62.48 ± 6.65	62.05 ± 6.61	8/13	7/14	–	–	KT	SI	1	–	①
Kaya Mutlu E 2017 (78)	54.25 ± 6.01	57.10 ± 6.26	4/16	2/17	–	–	KT	SI	3	4	①③④
Dogan N 2022 (79)	56.9 ± 6.9	55.7 ± 6.9	27	30	51.6 ± 38.0mon	41.7 ± 37.3mon	KT	SI	3	4	①②③④
Li JF 2018 (80)	65.2 ± 4.6	65.2 ± 4.6	14/28	17/25	31.8 ± 3.9mon	32.5 ± 3.6mon	KT	SI	4	–	⑤⑥
Anandkumar S 2014 (81)	55.7 ± 5.8	55.9 ± 5.0	9/11	8/12	8.4 ± 1.5mon	8.4 ± 1.1mon	KT	SI	4	–	①
Donec V 2020 (82)	68.7 ± 9.9	70.6 ± 8.3	17/64	16/60	–	–	KT	SI	4	4	④
Günaydin ÖE 2022 (83)	49~72	49~72	22	18	–	–	KT	ESW	6	–	①
Xin R 2021 (84)	53.19 ± 4.13	53.78 ± 4.45	23/17	24/16	19.32 ± 3.13yr	19.41 ± 3.56yr	PRP	NOD	12	–	①④⑤⑥
Acosta-Olivo C 2014 (85)	————	————	21	21	–	–	PRP	NSAIDs	4	24	⑤⑥
Huang XH 2022 (86)	56.76 ± 4.97	56.13 ± 4.43	21/19	23/17	2.07 ± 0.89yr	2.12 ± 0.83yr	PRP	NOD	4	8	①④⑥
Li YJ 2022 (87)	54.61 ± 8.42	55.83 ± 9.48	14/20	12/17	–	–	PRP	ESW	5	–	①④
Chu JB 2022 (88)	53.9 ± 5.0	54.5 ± 5.1	123/185	127/175	–	–	PRP	SI	12	12, 24	①②③⑤⑥
Patel S 2013 (89)	53.11 ± 11.55	51.64 ± 9.22	11/16	6/17	–	–	PRP	SI	6	12, 24	①②③
Park YB 2021 (90)	60.6 ± 8.2	62.3 ± 9.6	16/39	8/47	–	–	PRP	NOD	6	12, 24	①②③⑤
Wang YC 2022 (91)	61.87 ± 5.46	63.00 ± 5.33	12/42	16/40	–	–	PRP	NOD	4	4, 12, 24	②③
Bennell KL 2021 (92)	62.2 ± 6.3	61.6 ± 6.6	59/85	60/84	2~12yr	2.5~10yr	PRP	SI	3	8	①
Reyes-Sosa R 2020 (93)	53.7 ± 8.58	52.8 ± 9.64	4/26	9/21	–	–	PRP	NSAIDs	4	4, 12, 24	①②③
Cole BJ 2017 (94)	55.9 ± 10.4	56.8 ± 10.5	28/21	20/30	–	–	PRP	NOD	3	6, 12, 24	①②⑤⑥
Görmeli G 2017 (95)	53.8 ± 13.4	53.5 ± 14.0	19/25	17/22	>4month	>4month	PRP	NOD	4	6, 12, 24	①
		52.8 ± 12.8		20/20		>4month		SI			
Li HT 2017 (96)	59.11 ± 16.83	60.23 ± 16.48	18/14	15/17	3.04 ± 0.71yr	3.01 ± 0.86yr	EA	NOD	5	4	①②③
Liu Y 2022 (97)	58 ± 7	62 ± 8	7/23	10/20	6.6 ± 3.2yr	5.8 ± 2.8yr	EA	NSAIDs	3	–	①⑤⑥
Ju ZY 2017 (98)	60 ± 10	64 ± 6	6/24	7/23	29.89 ± 29.74mon	32.74 ± 31.43mon	EA	NSAIDs	2	–	①②③⑤⑥
Zhu DY 2019 (99)	40~70	40~70	31/44	29/46	21.48mon	21.68mon	EA	NOD	4	–	①②③⑤⑥
Yang S 2022 (100)	58.26 ± 9.32	54.95 ± 11.26	69/154	65/160	9.0 ± 5.0yr	7.0 ± 6.5yr	EA	TT	2	4	①④
Wang Q 2022 (101)	62 ± 9	62 ± 7	9/21	12/18	49.03 ± 40.55mon	52.90 ± 51.74mon	EA	SI	8	8, 18	④
Wu MX 2010 (102)	53.7 ± 8.2	52.8 ± 7.5	60/63	59/63	12.4 ± 9.9mon	11.2 ± 9.5mon	EA	NOD	4	–	⑥
Lv ZT 2019 (103)	64.6 ± 10.2	63.7 ± 9.3	39/106	15/60	–	–	EA	SI	2	–	①
Tu JF 2021 (104)	62.7 ± 6.6	62.8 ± 7.6	32/119	40/106	6.0 ± 5.3yr	7.5 ± 6.1yr	EA	SI	8	8, 18	②③
Hei G 2020 (105)	50~75	50~75	49	49	6~62mon	6~62mon	OT	NOD	3	–	⑤⑥
Li WW 2021 (106)	68.49 ± 4.45	68.17 ± 4.26	25	25	5.63 ± 0.52yr	5.48 ± 0.61yr	OT	NOD	5	–	①⑤⑥
Meng T 2018 (107)	61.27 ± 8.31	61.80 ± 8.26	21/25	20/26	4.02 ± 1.53yr	3.97 ± 1.48yr	OT	NOD	5	4, 8, 12	①⑤⑥
Lopes de Jesus CC 2017 (108)	70.5 ± 7.2	69.5 ± 7.6	5/56	5/30	1~16yr	1~16yr	OT	SI	8	8	①②③
Duymus TM 2017 (109)	59.4 ± 5.7	60.4 ± 5.1	4/31	1/32	–	–	OT	PRP	4	4, 12, 24	①②③
		60.3 ± 9.1		1/33		–		NOD			
Babaei-Ghazani A 2018 (110)	59.65 ± 10.24	56.26 ± 7.88	7/24	3/28	5.61 ± 1.31yr	5.58 ± 1.54yr	OT	NOD	4	4, 12	①④
Raeissadat SA 2021 (111)	57.60 ± 6.1	56.09 ± 6.0	12/36	13/39	4.42 ± 2.1yr	4.44 ± 2.3yr	OT	PRP	3	8, 24	①②③
		57.91 ± 6.7		12/37		3.86 ± 1.6yr		NOD			

T, Treatment group; C, Control group; -, It was not mentioned; ESW, Extracorporeal shock wave; RF, Radiofrequency; AT, Acupotomy; LT, Laser therapy; TT, Tuina therapy; KT, Kinesio taping; EA, Electroacupuncture; PRP, Platelet-Rich Plasma; OT, Ozone therapy; NOD, Non-oral drug; NSAIDs, Nonsteroidal antiinflammatory drugs; SI, Sham intervention;① VAS; ②WOMAC Pain; ③WOMAC Function; ④ROM; ⑤IL-β; ⑥TNF-α.

**Table 2:** Introduction to non-pharmacological interventions for knee osteoarthritis.

Interventions	Abbreviation	Description
Extracorporeal shock wave	ESW	ESW is a kind of mechanical pulse pressure wave conducted by the physical mechanism medium (air or gas). The device converts the pulse sound wave generated by the air into a precise ballistic shock wave. Through the positioning and moving of the therapeutic probe, it can produce good therapeutic effect on the human tissue where the pain occurs more widely.
Radiofrequency	RF	RF technology mainly relies on radiofrequency therapy instrument with ablation and cutting functions, and the treatment mechanism is mainly thermal effect. When the radiofrequency current flows through the human tissue, due to the rapid changes in the electromagnetic field, the polar water molecules in the tissue move at high speed, generating heat (that is, endogenous heat effect), resulting in evaporation, drying, shrinkage and shedding of water inside and outside the cell, resulting in aseptic necrosis, so as to achieve the purpose of treatment.
Acupotomy	AT	AT is based on the theory of meridians in traditional Chinese medicine, and integrates the concept of accurate anatomy and treatment in modern medicine. While “needle” plays the role of dredging meridians and regulating qi and blood, it also organically combines the function of “knife” to release local adhesion and relieve tension, so as to achieve the purpose of treatment
Laser therapy	LT	Laser therapy is a form of physical therapy that uses the biological effects of laser to tissue muscles to treat diseases.
Tuina therapy	TT	The clinician uses his hands to act on the patient’s body surface, the injured part, the discomfort place, the specific acupoint, the painful place, the specific use of pushing, holding, pressing, rubbing, kneading, pinching, point, patting and other forms of various techniques and forces, in order to achieve the purpose of treating diseases.
Kinesio taping	KT	Kinesio taping is an ultra-thin breathable tape with elasticity that comes in different widths, colors and elasticity and can be cut into different shapes as needed to be applied to the skin, muscles and joints in need of treatment. Compared with traditional poultices or cloths, it greatly reduces skin irritation or maladjustment and allows the treatment site to move naturally.
Electroacupuncture	EA	EA is a method of preventing and treating disease by combining needle and electrical stimulation by passing a trace current close to the body’s bioelectricity through the needle tool after the needle has been inserted into the acupoint to obtain Qi.
Platelet-pich plasma injection	PRP	Platelet-rich plasma injection therapy is to extract 10 to 20 milliliters of blood from the patient, and then separate the platelet-rich plasma through a centrifuge, and then inject the extracted platelet concentration concentrated liquid into the injured site to achieve the purpose of treatment.
Ozone therapy	OT	Ozone therapy is to inject different concentrations of ozone into the lesion through joint cavity puncture to achieve the purpose of treatment.

### 3.3 Risk of bias assessments

**Figure 2:**
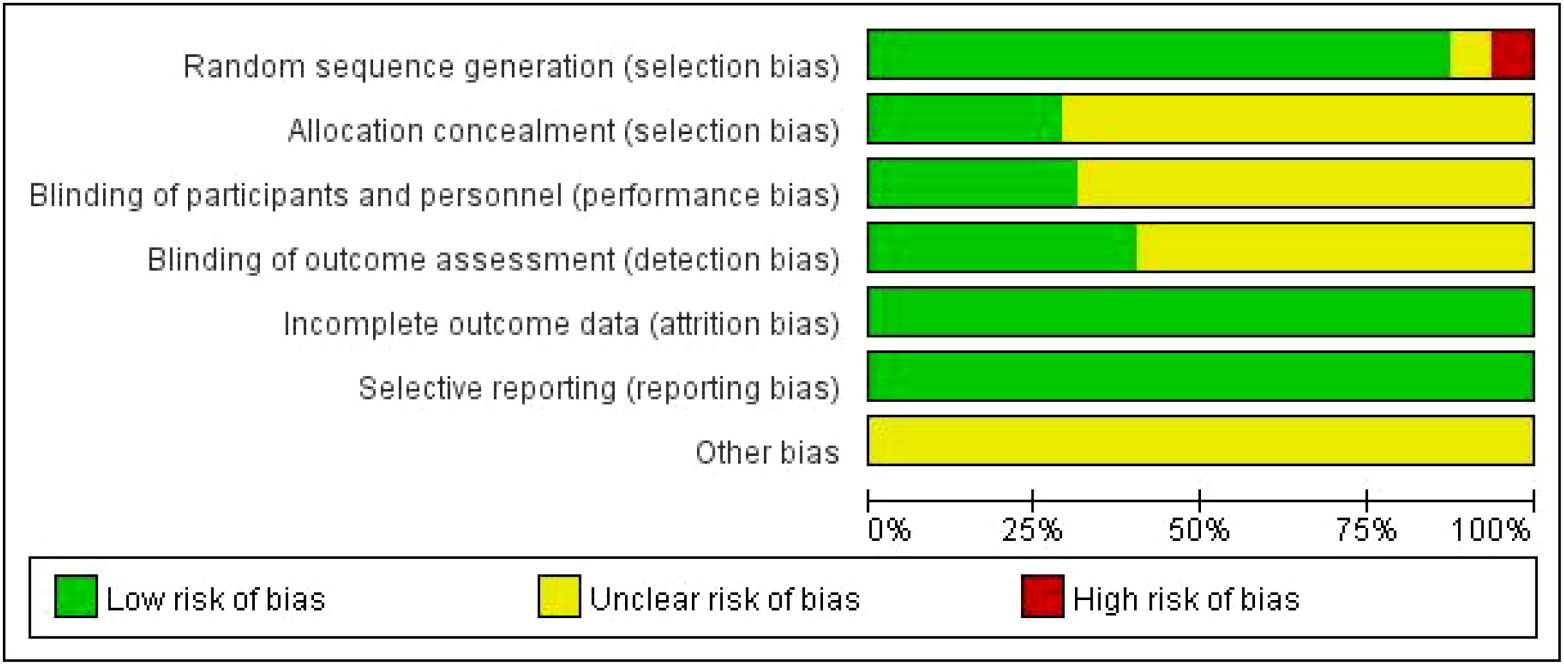
Literature bias evaluation results.

Of the 80 RCTs, 75 reported the generation of random sequences, whereas the remainder mentioned only random assignments. Thirty-three studies (34–36, 39, 41, 43–47, 53–56, 59–61, 64, 69, 71, 72, 75, 77, 80, 86, 89, 96, 98, 105–107) using random number tables, 21 (42, 49, 50, 63, 65, 66, 70, 74, 78, 82, 88, 90, 92–95, 100, 101, 103, 104, 109) using computer allocation randomization, 15 (40, 51, 57, 58, 67, 73, 79, 81, 83, 91, 97, 99, 108, 110, 111) using the envelope method, and one (102) study using the lottery method were all rated as low risk. Three (32, 33, 76) studies randomly selected patients according to different treatment methods, one (87) according to the patient’s wishes, and one (84) according to the order of admission, all of which were rated as high risk. Thirty-five studies mentioned blinding: 14 (40–42, 49, 50, 52, 57, 63, 73, 97, 101, 103, 110, 111) were single-blinded, 21 (43, 51, 58, 62, 64–66, 74, 78, 79, 81, 82, 88–92, 94, 95, 104, 108) were double-blinded, and 22 (40, 51, 57, 58, 62, 64, 66, 67, 73, 79, 81, 83, 91, 92, 94, 97, 99, 101, 103, 108, 110, 111) used allocation concealment rated as low risk. The remaining studies did not mention blinding or allocation concealment. All 80 studies reported the outcome indicators used in this study and did not identify falsified or incomplete data, with incomplete reporting and early discontinuation of trials rated as low risk. No other biases were mentioned in any study. The results are shown in **Figure 2**, while a summary of the risk of bias is shown in **Supplementary Figure S2**.

### 3.4 Network meta-analysis

The results of the heterogeneity test showed high heterogeneity for all outcome indicators (P < 0.05, I^2^ > 50%). Therefore, a random-effects model was used for all meta-analyses in this study. Except for long-term ROM, the evidence network diagrams of the outcome indicators were a closed loop. The node-splitting method showed good consistency with no heterogeneity emerging between the studies (P > 0.05). The results of the node-splitting tests are presented in **Supplementary Tables S2-10**.

#### 3.4.1 Short-term VAS

**Figure 3:**
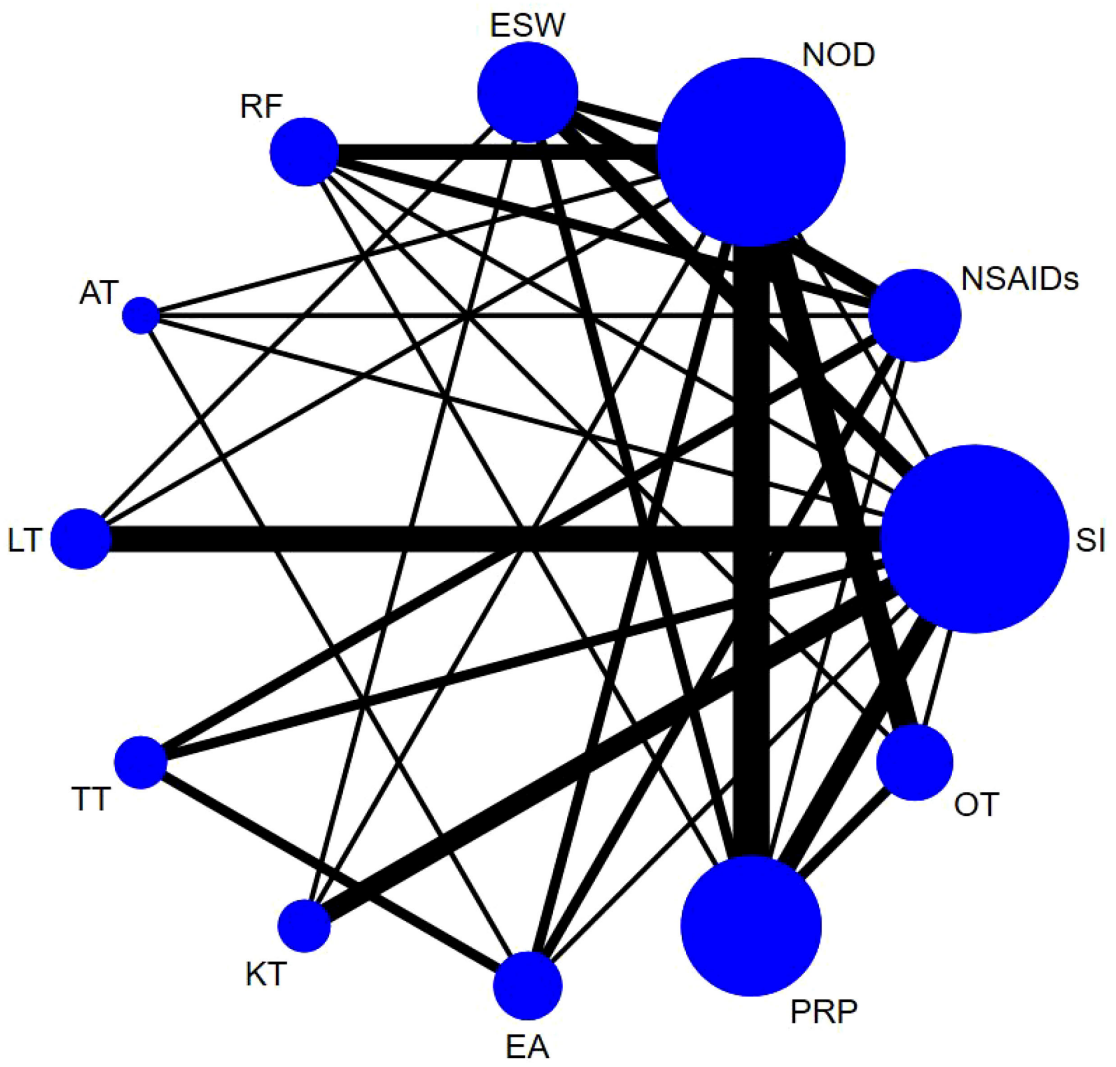
Evidence network diagram of VAS (short-term).

**Figure 4:**
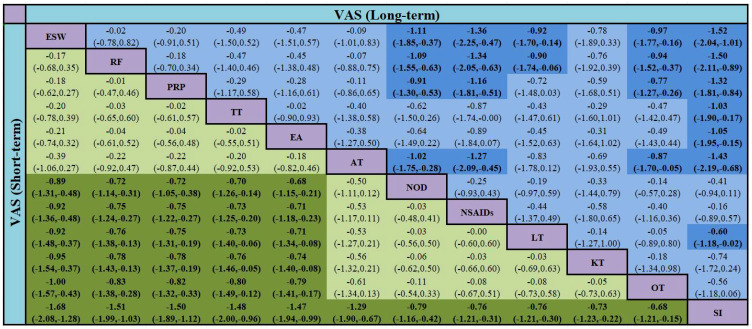
Network meta-analysis of VAS in short- and long-term [SMD(95% CI)].

Sixty-one studies (32–36, 39–49, 51–54, 58, 60–66, 68, 70, 72, 74–79, 81, 83, 84, 86–90, 92–100, 103, 106–111) involving 6,489 participants reported short-term post-treatment VAS scores. Twelve interventions were considered, and 66 two-by-two comparisons were performed. The evidence network generally centered on NOD, thereby forming 22 closed loops (**Figure 3**). Compared to SI, all non-drug and drug therapies had a better effect on the VAS score (P < 0.05). ESW, RF, PRP, TT, and EA significantly reduced the VAS score compared with NOD, NSAIDs, LT, KT, and OT (P < 0.05) (**Figure 4**).

**Figure 5:**
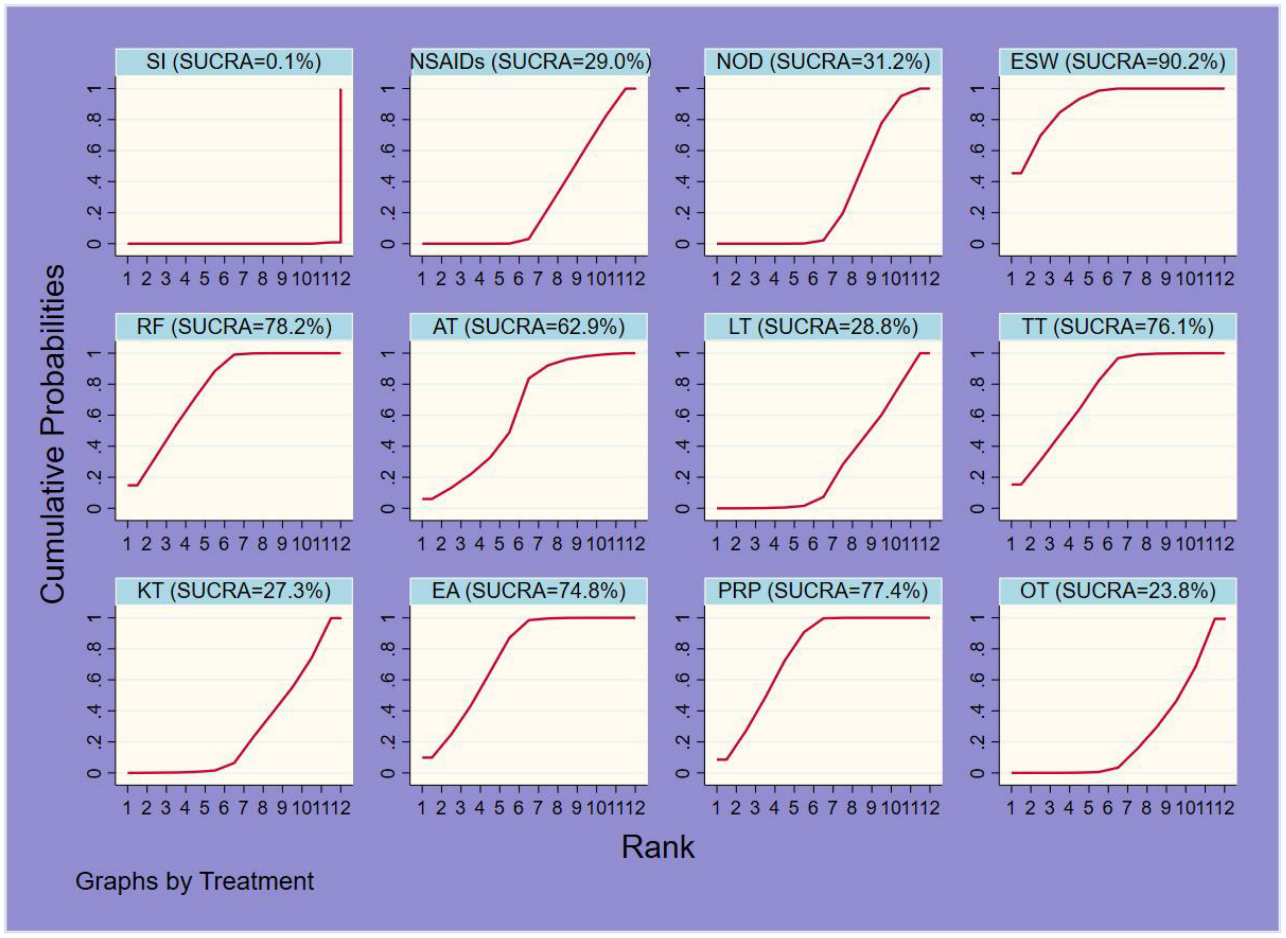
Ranking of SUCRA probabilities for short-term VAS.

**Figure 6:**
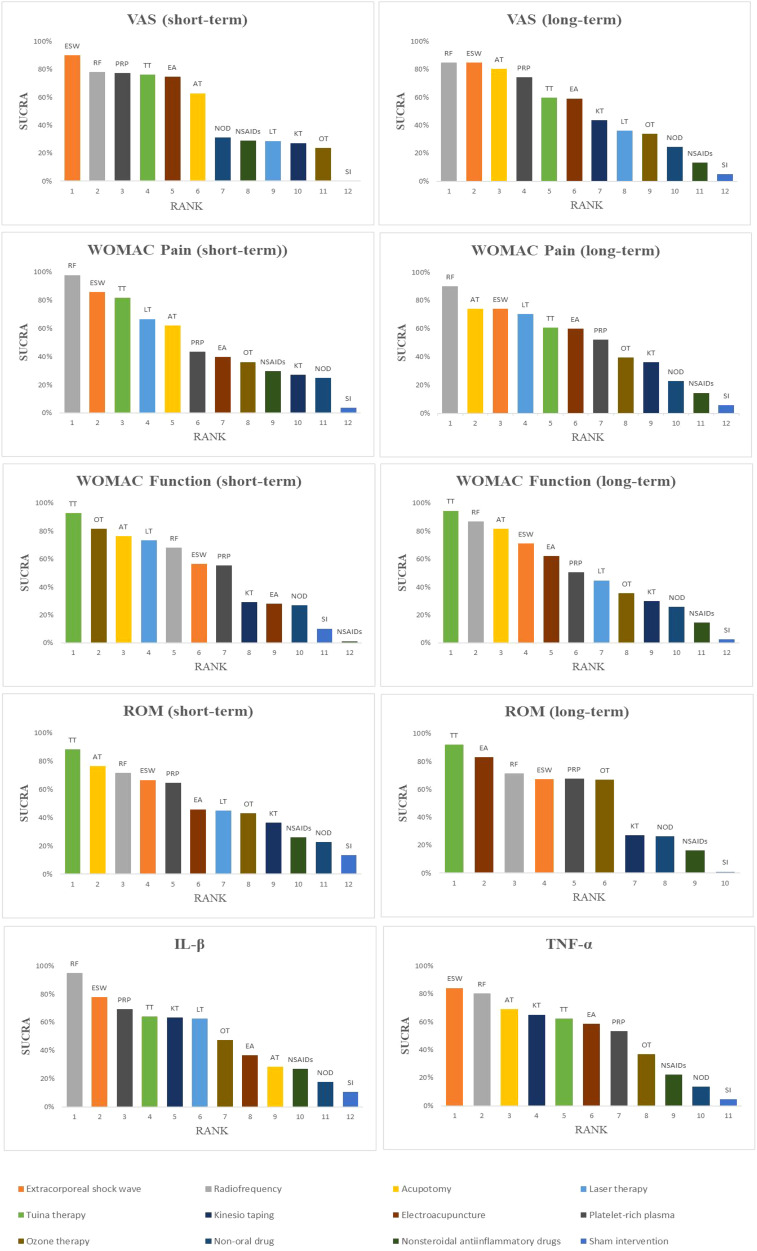
Ranking of SUCRA probabilities for each outcome indicator.

The probability ranking results of reducing short-term VAS were as follows: ESW (SUCRA=90.2%) > RF (78.2%) > PRP (77.4%) > TT (76.1%) > EA (74.8%) > AT (62.9%) > NOD (31.2%) > NSAIDs (29.0%) > LT (28.8%) > KT (27.3%) > OT (23.8%) > SI (0.1%) (**Figure 5**, **6**, **Table 3**).

**Table 3:** Ranking of SUCRA probabilities for each outcome indicator.

Intervention	VAS (short-term)		WOMAC Pain (short-term)		WOMAC Function (short-term)		ROM (short-term)		IL-β		
	SUCRA	RANK	SUCRA	RANK	SUCRA	RANK	SUCRA	RANK	SUCRA		RANK
ESW	90.2	1	85.9	2	56.6	6	66.7	4	77.7		2
RF	78.2	2	97.8	1	68.1	5	71.6	3	94.8		1
AT	62.9	6	62.1	5	76.4	3	76.4	2	28.6		9
LT	28.8	9	66.6	4	73.5	4	45.0	7	62.5		6
TT	76.1	4	81.7	3	92.8	1	88.3	1	64.2		4
KT	27.3	10	27.0	10	29.3	8	36.4	9	63.4		5
EA	74.8	5	39.7	7	28.0	9	45.9	6	36.7		8
PRP	77.4	3	43.5	6	55.3	7	64.8	5	69.4		3
OT	23.8	11	36.2	8	81.5	2	43.0	8	47.4		7
NOD	31.2	7	25.0	11	27.0	10	22.6	11	17.8		11
NSAIDs	29.0	8	29.6	9	1.3	12	26.0	10	26.9		10
SI	0.1	12	3.8	12	10.3	11	13.3	12	10.8		12
Intervention	VAS (long-term)		WOMAC Pain (long-term)		WOMAC Function (long-term)		ROM (long-term)		TNF-α		
	SUCRA	RANK	SUCRA	RANK	SUCRA	RANK	SUCRA	RANK	SUCRA	RANK	
ESW	84.7	2	73.9	3	71.2	4	67.3	6	84.2	1	
RF	84.8	1	90.1	1	86.9	2	71.4	3	80.2	2	
AT	80.4	3	74.2	2	81.7	3	–	–	69.2	3	
LT	36.1	8	70.4	4	44.7	7	–	–	–	–	
TT	59.9	5	60.8	5	94.5	1	92.1	1	62.3	5	
KT	43.7	7	36.2	9	30.1	9	27.3	7	65.1	4	
EA	59.2	6	59.9	6	62.1	5	83.0	2	58.5	6	
PRP	74.5	4	52.0	7	50.5	6	67.6	4	53.3	7	
OT	33.9	9	39.6	8	35.4	8	67.0	5	36.9	8	
NOD	24.5	10	22.7	10	25.8	10	26.5	8	13.6	10	
NSAIDs	13.3	11	14.5	11	14.5	11	16.4	9	22.2	9	
SI	5.0	12	5.7	12	2.6	12	1.1	10	4.6	11	

The bold font indicates that there was a statistically significant difference between the two treatments.

#### 3.4.2 Long-term VAS

**Figure 7:**
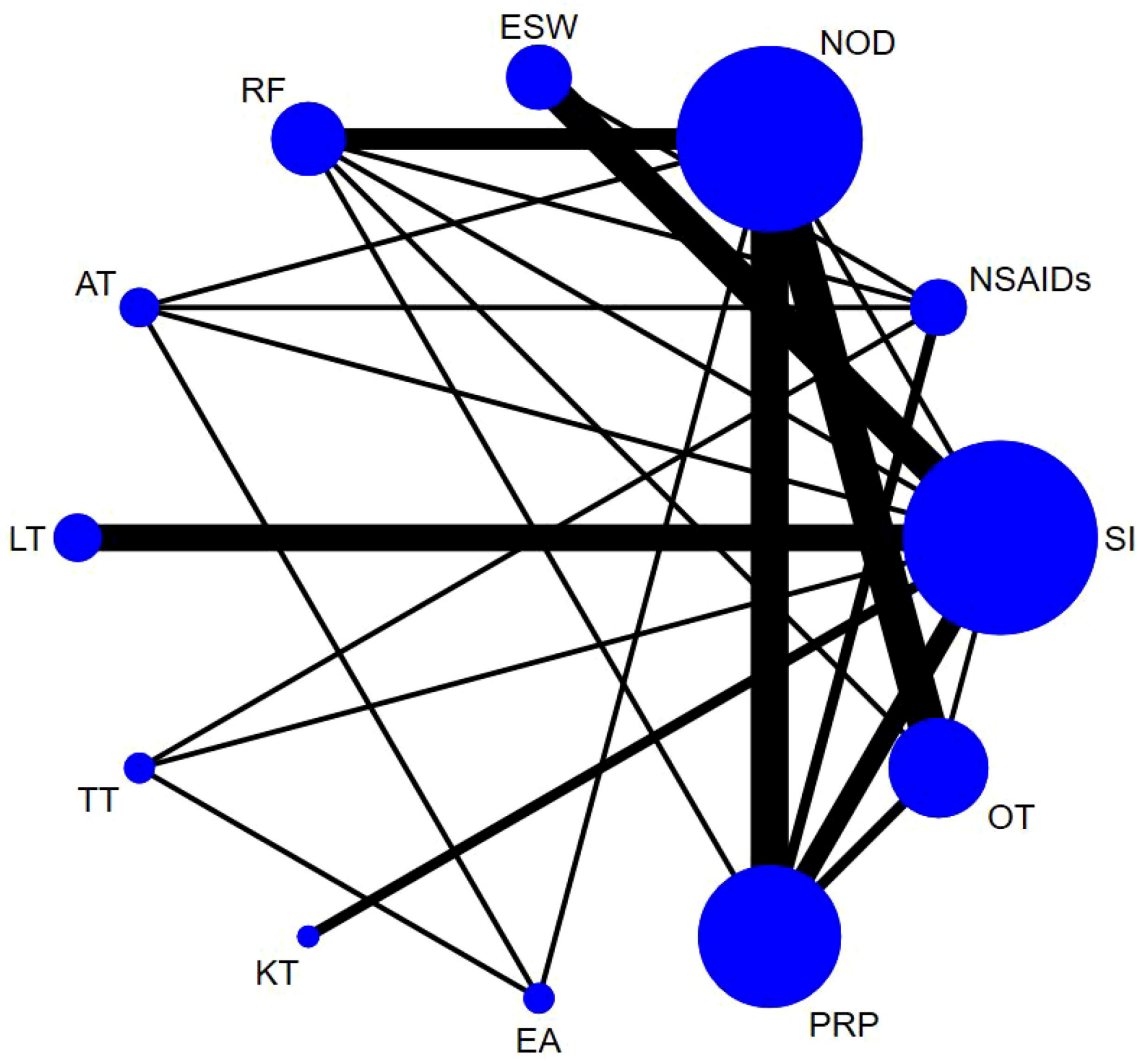
Evidence network diagram of VAS (long-term).

Thirty-six studies (39, 41–46, 49, 51–54, 58, 60, 62, 64, 66, 68, 74, 78, 79, 81, 83, 84, 86, 88–90, 92–96, 100, 107–111), involving 5,925 participants reported long-term VAS score. Twelve interventions were considered and 66 two-by-two comparisons were performed. The overall evidence network centered on SI, thereby forming 14 closed loops (**Figure 7**). The results of the meta-analysis indicated that compared with LT, RF (SMD = -0.90, 95% CI [-1.74, -0.06]) and ESW (SMD = -0.92, 95% CI [-1.70, -0.14]) significantly reduced the VAS score. RF, ESW, AT, and PRP were superior to OT, NOD, NSAIDs, and SI in terms of performance. Compared to SI, TT, EA, and LT were more effective in decreasing the long-term VAS scores. All of the above-mentioned differences were statistically significant (P < 0.05) (**Figure 4**).

The probability ranking results for reducing the long-term VAS scores were as follows: RF (84.8%) > ESW (84.7%) > AT (80.4%) > PRP (74.5%) > TT (59.9%) > EA (59.2%) > KT (43.7%) > LT (36.1%) > OT (33.9%) > NOD (24.5%) > NSAIDs (13.3%) > SI (5.0%) (**Figure 6**, **Table 3**).

#### 3.4.3 WOMAC pain (short-term)

Thirty-six studies (37, 38, 41–43, 50, 52, 54–56, 58, 60, 62–64, 66–70, 73, 74, 79, 88–91, 93, 94, 96, 98, 99, 104, 108, 109, 111) involving 4207 patients investigated short-term WOMAC pain score. Twelve types of interventions were considered, and 66 two-by-two comparisons were performed. The evidence network was generally centered on the SI, thereby forming 12 closed loops (**Supplementary Figure S3**). Compared with OT, RF [SMD = -4.79, 95% CI (-7.98, -1.60)] and ESW [SMD = -2.93, 95% CI (-5.59, -0.26)] significantly reduced the short-term WOMAC pain score (P < 0.05). RF, ESW, and TT were better interventions than PRP, EA, NSAIDs, NOD, and SI (P < 0.05). Compared with AT and KT, RF was more effective in reducing the WOMAC pain score (P < 0.05). LT, AT, PRP, and EA were better than SI (P < 0.05) (**Supplementary Figure S11**).

RF (97.8%) may be the most effective intervention in improving the short-term WOMAC pain score, followed by ESW (85.9%), TT (81.7%), LT (66.6%), AT (62.1%), PRP (43.5%), EA (39.7%), OT (36.2%), NSAIDs (29.6%), KT (27.0%), NOD (25.9%), and SI (3.8%) (**Figure 6**, **Table 3**).

#### 3.4.4 WOMAC pain (long-term)

Twenty-nine studies (41–43, 50, 52, 54, 55, 58, 60, 62, 64, 66, 68, 69, 73, 74, 79, 88–91, 93, 94, 96, 104, 108, 109, 111) involving 4804 patients investigated long-term WOMAC pain score. Twelve types of interventions were considered and 66 two-by-two comparisons were performed. The evidence network was generally centered on SI, thereby forming eight closed loops (**Supplementary Figure S4**). Compared with NOD, RF [SMD = -4.70, 95% CI (-7.60, -1.81)], AT [SMD = -3.22, 95% CI (-6.13, -0.32)], and ESW [SMD = -3.19, 95% CI (-6.36, -0.02)] significantly reduced the long-term WOMAC pain score. RF, AT, ESW, LT, TT, and PRP were better interventions than NSAIDs and SI. RF was a superior intervention to OT. Compared with SI, EA was more effective in decreasing long-term WOMAC pain scores. All the above-mentioned differences were statistically significant (P < 0.05) (**Supplementary Figure S11**).

RF may be the most effective therapy for long-term WOMAC pain score reduction (90.1%), followed by AT (74.2%), ESW (73.9%), LT (70.4%), TT (60.8%), EA (59.9%), PRP (52.0%), OT (39.6%), KT (36.2%), NOD (22.7%), NSAIDs (14.5%), and SI (5.7%) (**Figure 6**, **Table 3**).

#### 3.4.5 WOMAC function (short-term)

Thirty-seven studies (37, 38, 41–43, 50, 52–56, 58, 60, 62–64, 66–70, 73, 74, 78, 79, 88–91, 93, 96, 98, 99, 104, 108, 109, 111) involving 4,207 participants reported short-term WOMAC function score. Twelve interventions were considered and 66 two-by-two comparisons were performed. The overall evidence network centered on SI, thereby forming 14 closed loops (**Supplementary Figure S5**). Except for KT, all non-drug therapies and NOD were superior to NSAIDs in reducing short-term WOMAC function (P < 0.05). Compared to NOD and SI, TT, OT, AT, LT, RF, ESW, and PRP were more effective in decreasing WOMAC function (P < 0.05). TT was a better intervention than PRP, KT, and EA (P < 0.05). OT, AT, and LT significantly reduced the WOMAC function score compared with EA (P < 0.05) (**Supplementary Figure S12**).

The probability ranking results of improving short-term WOMAC function scores were as follows: TT (92.8%) > OT (81.5%) > AT (76.4%) > LT (73.5%) > RF (68.1%) > ESW (56.6%) > PRP (55.3%) > KT (29.3%) > EA (28.0%) > NOD (27.0%) > SI (10.3%) > NSAIDs (1.3%) (**Figure 6**, **Table 3**).

#### 3.4.6 WOMAC function (long-term)

Twenty-nine studies (41–43, 50, 52–55, 58, 60, 62, 64, 66, 68, 69, 73, 74, 78, 79, 88–91, 93, 96, 104, 108, 109, 111) involving 4705 patients investigate long-term WOMAC function. Twelve types of interventions were considered, and 66 two-by-two comparisons were performed. The evidence network was generally centered on the SI, thereby forming eight closed loops (**Supplementary Figure S6**). The results of the meta-analysis indicated that except for KT, all non-drug therapies and NOD were superior to SI in improving long-term WOMAC function (P < 0.05). Compared with NOD and NSAIDs, TT, RF, AT, and PRP were more effective in reducing WOMAC function scores (P < 0.05). AT was superior to PRP [SMD = -4.28, 95% CI (-8.49, -0.07)] and OT [SMD = -5.92, 95% CI (-10.96, -0.89)] (P < 0.05). TT and RF were superior to PRP, OT, and KT. Compared to LT, TT significantly reduced WOMAC function (P < 0.05) (**Supplementary Figure S12**).

TT (94.5%) may be the most effective intervention in reducing the long-term WOMAC function score, followed by RF (86.9%), AT (81.7%), ESW (71.2%), EA (62.1%), PRP (50.5%), LT (44.7%), OT (35.4%), KT (30.1%), NOD (25.8%), NSAIDs (14.5%), and SI (2.6%) (**Figure 6**, **Table 3**).

#### 3.4.7 ROM (short-term)

Twenty-one studies (36, 37, 42, 45, 51, 58, 59, 61, 65, 68, 70, 74, 76, 78, 79, 82, 84, 86, 87, 100, 101, 110) involving 2369 patients reported on short-term ROM. Twelve types of interventions were considered and 66 two-by-two comparisons were performed. The evidence network was generally centered on the SI, thereby forming four closed loops (**Supplementary Figure S7**). Compared to SI, TT [SMD = 14.65, 95% CI (6.70, 22.60)], AT [SMD = 12.29, 95% CI (1.66, 22.93)], RF [SMD = 11.10, 95% CI (0.44, 21.75)], and ESW [SMD = 9.91, 95% CI (1.24, 18.58)] were more effective in increasing short-term ROM. TT was a better intervention than KT, NSAIDs, or NOD (P < 0.05) (**Supplementary Figure S13**).

TT (88.3%) may be the most effective treatment for improving short-term ROM, followed by AT (76.4%), RF (71.6%), ESW (66.7%), PRP (64.8%), EA (45.9%), LT (45.0%), OT (43.0%), KT (36.4%), NSAIDs (26.0%), NOD (22.6%), and SI (13.3%) (**Figure 6**, **Table 3**).

#### 3.4.8 ROM (long-term)

Thirteen studies (42, 45, 51, 58, 68, 74, 78, 79, 82, 86, 100, 101, 110) involving 1372 participants and 10 interventions reported long-term ROM as an outcome measure. Thus, 45 two-by-two comparisons were made, with an overall evidence network centered on the SI (**Supplementary Figure S8**). The results of the NMA indicated that all non-pharmacological interventions and NOD were superior to SI in improving long-term ROM (P < 0.05). Compared to NOD, PRP (SMD = 5.07, 95% CI (1.62, 8.52)] significantly increased the long-term ROM (P < 0.05). TT was a better intervention than ESW [SMD = 5.40, 95% CI (1.13, 9.66)] (P < 0.05). Compared with KT, NOD, and NSAIDs, TT, EA, RF, and OT were more effective in improving the long-term ROM (**Supplementary Figure S13**).

The probability ranking results for improving long-term ROM were as follows: TT (92.1%) > EA (83.0%) > RF (71.4%) > PRP (67.6%) > OT (67.0%) > ESW (47.3%) > KT (27.6%) > NOD (26.5%) > NSAIDs (16.4%) > SI (1.1%) (**Figure 6**, **Table 3**).

#### 3.4.9 IL-1β

Twenty-three studies (32–34, 44, 48, 56, 57, 59, 64, 71, 72, 80, 84, 85, 88, 90, 94, 97–99, 105–107) involving 2674 patients investigated IL-1β levels. Twelve types of interventions were considered, and 66 two-by-two comparisons were performed. The evidence network was generally centered on NOD, thereby forming three closed loops (**Supplementary Figure S9**). RF, ESW, and PRP were better interventions than NSAIDs, NOD, and SI. Compared to OT [SMD = -15.14, 95% CI (-27.74, -2.55)], EA [SMD = -17.61, 95% CI (-31.58, -3.64)], and OT [SMD = -19.28, 95% CI (-32.96, -5.60)], RF significantly decreased IL-1β levels (P < 0.05). ESW significantly reduced IL-1β levels compared with AT (P < 0.05) (**Supplementary Figure S14**).

The results showed that RF (94.8%) may be the most effective therapy for reducing IL-1β levels, followed by ESW (77.7%), PRP (69.4%), TT (64.2%), KT (63.4%), LT (62.5%), OT (47.4%), EA (36.7%), AT (28.6%), NSAIDs (26.9%), NOD (17.8%), and SI (10.8%) (**Figure 6**, **Table 3**).

#### 3.4.10 TNF-α

Twenty-seven studies (32, 33, 35, 39, 44, 46, 47, 53, 55–57, 59, 72, 75, 80, 84–86, 88, 94, 97–99, 102, 105–107) involving 2825 patients assessed TNF-α levels. Eleven types of interventions were considered, and 55 two-by-two comparisons were performed. The evidence network was generally centered on NOD, thereby forming four closed loops (**Supplementary Figure S10**). The NMA results showed that except for OT, all non-drug therapies were superior to SI in reducing TNF-α levels (P < 0.05). Compared to NOD, ESW, RF, AT, EA, and PRP significantly improved TNF-α levels (P < 0.05). ESW, RF, and AT were better than NSAIDs (P < 0.05) (**Supplementary Figure S14**).

ESW (84.2%) may be the most effective intervention in reducing TNF-α levels, followed by RF (80.2%), AT (69.2%), KT (65.1%), TT (62.3%), EA (58.5%), PRP (53.3%), OT (36.9%), NSAIDs (22.2%), NOD (13.6%), and SI (4.6%) (**Figure 6**, **Table 3**).

### 3.5 Publication bias

The indicators included in this study were tested for publication bias (**Supplementary Figure S15-24**). The indicators for WOMAC pain (long-term) and TNF-α were asymmetric in the funnel plots, suggesting a publication bias or small sample effect, which may have affected the results of the corresponding indicators. The funnel plots for the other indicators were symmetrical, suggesting a low possibility of publication bias in the current study.

### 3.6 Sensitivity analysis

To test the stability and reliability of the NMA results, we performed sensitivity analyses for short- and long-term VAS and WOMAC pain and function. First, five papers that were evaluated as high-risk in terms of literature quality were excluded (32, 33, 76, 84, 87), and sensitivity analyses were performed before and after exclusion. Second, as RCTS with small sample sizes may have affected the accuracy of the results, 11 studies with sample sizes of <50 were excluded from the sensitivity analysis (40, 44, 45, 61, 63, 67, 77, 78, 81, 83, 85).. The results showed little difference between the results before and after the exclusion of the two sensitivity analyses, indicating that the quality of the literature was good and that the results of the network meta-analysis were solid and stable. The results of the sensitivity analysis are shown in **Supplementary Figure S25-31** and **Table S11**.

### 3.7 Subgroup analysis

To reduce heterogeneity caused by inconsistent treatment and follow-up cycles, two subgroup analyses were performed. First, the study population was divided into two subgroups according to the treatment duration (<4 weeks and ≥4 weeks). Second, the follow-up period was divided into two subgroups (<12 weeks and ≥12 weeks). Regarding the outcomes of this analysis, only NMA of partial outcomes (VAS, WOMAC pain, and function) could be performed.

The rankings of non-pharmacological therapies showed little variation between VAS and WOMAC pain, whereas the rankings of pharmacological therapies varied considerably. For treatment courses <4 weeks, the efficacy of the NOD and NSAIDs was better than that of some non-drug therapies; however, for treatment courses ≥4 weeks, the effect was inferior to all non-drug therapies. The <12-week and ≥12-week follow-up subgroups showed no significant difference.

In terms of WOMAC function, the comparison between the <4-week and ≥4-week subgroups showed that OT lost its ranking advantage over AT and LT, and no significant difference in the other comparisons. The NMA results and rankings for subgroup analyses based on the follow-up period remained consistent with those before subgrouping. The results of the subgroup analysis are shown in **Supplementary Figure S32-34** and **Table S12**.

## 4 Discussion

There remains no consensus regarding the use of non-pharmacological interventions for the treatment of KOA. This study conducted NMA to generate a hierarchy of treatment rankings (112). The ranking probabilities for these treatment plans were calculated in terms of their clinical efficacy and inflammatory cytokine levels at various endpoints to provide a basis for making optimal choices.

This study included 80 RCTs that adopted nine non-drug interventions and included a total of 8440 individuals. VAS and WOMAC pain scores were used as pain indicators, while WOMAC function and ROM were used as functional indicators to evaluate the effect of non-pharmacological treatments on the improvement of short- and long-term symptoms in patients with KOA. The results of the NMA demonstrated that each non-drug therapy was superior to the sham intervention in terms of improving all efficacy indicators. Except for the short-term VAS and WOMAC pain outcomes, all non-drug therapies showed better efficacy than pharmacological treatments. An in-depth analysis of the indicators revealed that the immediate analgesic effect of NODs and NSAIDs was significant and superior to that of some nonpharmacological therapies, while their long-term analgesic efficacy was inferior to that of all nonpharmacological therapies. This may be related to drug resistance and other bottlenecks. Moreover, the short- and long-term effects of these drugs on improving joint function are poor. For short-term VAS, ESW therapy (90.2%) had the greatest likelihood of achieving the best efficacy among the treatment regimens, followed by RF therapy (78.2%) and PRP injection (77.4%). Radiofrequency therapy (84.8%, 97.8%, and 90.1%, respectively) may be the most promising method for reducing long-term VAS and short- and long-term WOMAC pain scores (84.8%, 97.8%, and 90.1%, respectively). Both ESW and RF were effective in improving short-term pain symptoms; however, radiofrequency was more effective for long-term analgesia. RF therapy may be a better choice in both the short and long term. The reason for the better effect of RF may be the inhibition of pain-sensing nerve C fibers and the promotion of endogenous opioid precursor mRNA transcription and related opioid peptide production (113, 114). TT can effectively improve WOMAC function and ROM in the short and long term, indicating that it can be given priority for treatment in patients with symptoms of functional dysfunction. Moreover, TT can regulate the interaction between interleukin-1β and the ERK1/2-nuclear transcription factor κB signaling pathway, thereby inhibiting excessive chondrocyte apoptosis, maintaining the stability of the internal environment of chondrocytes, and repairing injured cartilage tissue to restore functional activities of patients (115).

Inflammatory cytokines are important for maintaining the homeostasis of the internal environment of the knee (116). The representative inflammatory cytokines IL-β and TNF-α participate in chondrocyte apoptosis and proliferation and are closely related to KOA occurrence and development (117, 118). Additionally, the secretion of inflammatory factors is closely associated with pain symptoms (119). Our analysis of the levels of two outcome indicators, IL-β and TNF-α, showed that ESW and RF, which ranked higher in pain indicators, could also more effectively improve IL-β and TNF-α levels and showed a positive correlation, which may be the basis of its mechanism of action. Improvements in functional indicators correlated poorly with changes in inflammatory cytokine levels.

Our assessment of the quality of the literature showed that almost all studies used low-risk random assignment methods. The literature included in previous studies rarely mentioned blinding and allocation concealment, or described them inaccurately. In the present analysis, nearly half of the studies explicitly proposed blinded methods and allocation concealment and described specific implementation methods. Part of the study adopted multi-center and large-sample research, which improved its credibility. To enhance the strength of the evidence, we conducted two sensitivity analyses of the VAS: WOMAC pain and WOMAC function (short- and long-term). After excluding high-risk and small-sample studies, the overall results remained robust, indicating that the quality of the included literature was acceptable.

The results of subgroup analysis showed no significant difference between the <12-week and ≥12-week follow-up subgroups. The treatment course subgroups showed little change in the ranking of non-drug treatments, whereas there was a greater change in the ranking of drug treatments in VAS and WOMAC pain. For treatment courses <4 weeks, the efficacy of NOD and NSAIDs was better than that of some non-drug therapies; in contrast, for treatment courses ≥4 weeks, the effect was inferior to all non-drug therapies. This finding indicated that the immediate analgesic effect of the drug was better and the long-term effect was worse, which is consistent with the above follow-up ranking results. In terms of WOMAC function, the comparison between the <4-week and ≥4-week subgroups showed OT lost its ranking advantage over AT and LT, which may be related to the slow onset of AT and LT (120).

However, this study has some limitations. First, during literature selection in the present study, not all existing literature could be included because the original text for some studies could not be found and some studies used geometric means. Second, the sample sizes of the included studies were limited, which might have affected the accuracy of the results. Second, fewer studies in this analysis published pretrial protocols, which may have led to selective reporting bias. Finally, other non-drug therapies, such as wedge insoles and pulsed ultrasound, were lacking owing to the limited number of original studies, thus preventing the comparison of the effects of all non-pharmacological interventions.

## 5 Conclusion

The results of the comprehensive comparison of the outcome indicators of 9 different EANPI showed that radiofrequency was effective in relieving pain, and that tuina therapy can be given priority for treatment in patients with hypofunction as their main symptom. In clinical practice, an appropriate treatment method should be selected based on the actual situation. EANPI to improve pain symptoms may be related to the regulation of inflammatory cytokine levels, which may be a potential mechanism of action. Owing to the limitation of the quality and quantity of the included studies, more large-sample, multi-center, high-quality RCT studies are needed to verify our conclusions.

## Data availability statement

The original contributions presented in the study are included in the article/**Supplementary Material**. Further inquiries can be directed to the corresponding author.

## Author contributions

ZhenW: Methodology, Software, Visualization, Writing – original draft, Writing – review & editing. HX: Funding acquisition, Investigation, Project administration, Writing – original draft, Writing – review & editing. ZhengW: Data curation, Formal Analysis, Investigation, Project administration, Supervision, Writing – review & editing. HZ: Conceptualization, Methodology, Project administration, Writing – review & editing. JD: Conceptualization, Formal Analysis, Visualization, Writing – review & editing. LZ: Formal Analysis, Investigation, Methodology, Writing – review & editing. YW: Conceptualization, Software, Supervision, Validation, Writing – review & editing. ML: Methodology, Software, Supervision, Writing – review & editing. YZ: Investigation, Project administration, Resources, Supervision, Writing – original draft, Writing – review & editing.
