# Supplementary material for: Effects of externally-applied, non-pharmacological Interventions on short- and long-term symptoms and inflammatory cytokine levels in patients with knee osteoarthritis: a systematic review and network meta-analysis
Source: Front Immunol. 2023 Dec 14;14:1309751. doi: 10.3389/fimmu.2023.1309751 (PMC10752972; doi:10.3389/fimmu.2023.1309751)
Supplement: Supplementary file 1 [file DataSheet_1.pdf]

Effect of Externally-applied, Non-pharmacological Interventions on Short- and Long-term Efficacy and Inflammatory Cytokine Levels in Patients with Knee Osteoarthritis:A Systematic Review and Network Meta-Analysis

Supplementary materials

PRISMA-NMA checklist	Table S1	Page 2-6
Search Strategies of Pubmed	Figure S1	Page 7
Risk of bias summary	Figure S2	Page 8
Node-splitting test	Table S2-10	Page 9-14
Evidence network diagram	Figure S3-10	Page 15-18
The results of network meta-analysis	Figure S11-14	Page 19-20
Funnel plot	Figure S15-24	Page 21-23
The results of sensitivity analysis	Table S11<br>Figure S25-31	Page 24-28
The results of subgroup analysis	Table S12<br>Figure S32-34	Page 29-32

Table S1 PRISMA NMA Checklist

Section/Topic	Item #	Checklist Item	Reported on Page #
**TITLE**			
Title	1	Identify the report as a systematic review *incorporating a network meta-analysis (or related form of meta-analysis).*	**1**
**ABSTRACT**			
Structured summary	2	Provide a structured summary including, as applicable:<br>**Background:** main objectives<br>**Methods:** data sources; study eligibility criteria, participants, and interventions; study appraisal; and *synthesis methods, such as network meta-analysis.*<br>**Results:** number of studies and participants identified; summary estimates with corresponding confidence/credible intervals; *treatment rankings may also be discussed. Authors may choose to summarize pairwise comparisons against a chosen treatment included in their analyses for brevity.*<br>**Discussion/Conclusions:** limitations; conclusions and implications of findings.<br>**Other:** primary source of funding; systematic review registration number with registry name.	**1**
**INTRODUCTION**			
Rationale	3	Describe the rationale for the review in the context of what is already known*, including mention of why a network meta-analysis has been conducted.*_	**2**
Objectives	4	Provide an explicit statement of questions being addressed, with reference to participants, interventions, comparisons, outcomes, and study design (PICOS).	**3**
**METHODS**			
Protocol and registration	5	Indicate whether a review protocol exists and if and where it can be accessed (e.g., Web address); and, if available, provide registration information, including registration number.	**3**
Eligibility criteria	6	Specify study characteristics (e.g., PICOS, length of follow-up) and report characteristics (e.g., years	**3**

		considered, language, publication status) used as criteria for eligibility, giving rationale. *Clearly describe eligible treatments included in the treatment network, and note whether any have been clustered or merged into the same node (with justification).*_	
Information sources	7	Describe all information sources (e.g., databases with dates of coverage, contact with study authors to identify additional studies) in the search and date last searched.	**3**
Search	8	Present full electronic search strategy for at least one database, including any limits used, such that it could be repeated.	**SM 7**
Study selection	9	State the process for selecting studies (i.e., screening, eligibility, included in systematic review, and, if applicable, included in the meta-analysis).	**4**
Data collection process	10	Describe method of data extraction from reports (e.g., piloted forms, independently, in duplicate) and any processes for obtaining and confirming data from investigators.	**4**
Data items	11	List and define all variables for which data were sought (e.g., PICOS, funding sources) and any assumptions and simplifications made.	**4, 17**
**Geometry of the network**	**S1**	Describe methods used to explore the geometry of the treatment network under study and potential biases related to it. This should include how the evidence base has been graphically summarized for presentation, and what characteristics were compiled and used to describe the evidence base to readers.	
Risk of bias within individual studies	12	Describe methods used for assessing risk of bias of individual studies (including specification of whether this was done at the study or outcome level), and how this information is to be used in any data synthesis.	**4**
Summary measures	13	State the principal summary measures (e.g., risk ratio, difference in means). *Also describe the use of additional summary measures assessed, such as treatment rankings and surface under the cumulative ranking curve (SUCRA) values, as well as modified approaches used to present summary findings from meta-analyses.*	**4-5**
Planned methods of analysis	14	Describe the methods of handling data and combining results of studies for each network meta-analysis. This should include, but not be	**4**

		limited to:<br>• *Handling of multi-arm trials;*<br>• *Selection of variance structure;*<br>• *Selection of prior distributions in Bayesian analyses; and*<br>• *Assessment of model fit.*	
**Assessment of Inconsistency**	**S2**	Describe the statistical methods used to evaluate the agreement of direct and indirect evidence in the treatment network(s) studied. Describe efforts taken to address its presence when found.	**4-5**
Risk of bias across studies	15	Specify any assessment of risk of bias that may affect the cumulative evidence (e.g., publication bias, selective reporting within studies).	**4-5**
Additional analyses	16	Describe methods of additional analyses if done, indicating which were pre-specified. This may include, but not be limited to, the following:<br>• Sensitivity or subgroup analyses;<br>• Meta-regression analyses;<br>• *Alternative formulations of the treatment network; and*<br>• *Use of alternative prior distributions for Bayesian analyses (if applicable).*_	**4, 5, 15**
**RESULTS†**			
Study selection	17	Give numbers of studies screened, assessed for eligibility, and included in the review, with reasons for exclusions at each stage, ideally with a flow diagram.	**5**
**Presentation of network structure**	**S3**	Provide a network graph of the included studies to enable visualization of the geometry of the treatment network.	**9, 11, SM16-19**
**Summary of network geometry**	**S4**	Provide a brief overview of characteristics of the treatment network. This may include commentary on the abundance of trials and randomized patients for the different interventions and pairwise comparisons in the network, gaps of evidence in the treatment network, and potential biases reflected by the network structure.	**8-14**
Study	18	For each study, present characteristics for which	**5-8**

characteristics		data were extracted (e.g., study size, PICOS, follow-up period) and provide the citations.	
Risk of bias within studies	19	Present data on risk of bias of each study and, if available, any outcome level assessment.	**8, SM 9**
Results of individual studies	20	For all outcomes considered (benefits or harms), present, for each study: 1) simple summary data for each intervention group, and 2) effect estimates and confidence intervals. *Modified approaches may be needed to deal with information from larger networks.*	**8-14**
Synthesis of results	21	Present results of each meta-analysis done, including confidence/credible intervals. *In larger networks, authors may focus on comparisons versus a particular comparator (e.g. placebo or standard care), with full findings presented in an appendix. League tables and forest plots may be considered to summarize pairwise comparisons.* If additional summary measures were explored (such as treatment rankings), these should also be presented.	**8-14**
**Exploration for inconsistency**	**S5**	Describe results from investigations of inconsistency. This may include such information as measures of model fit to compare consistency and inconsistency models, *P* values from statistical tests, or summary of inconsistency estimates from different parts of the treatment network.	**8-9**
Risk of bias across studies	22	Present results of any assessment of risk of bias across studies for the evidence base being studied.	
Results of additional analyses	23	Give results of additional analyses, if done (e.g., sensitivity or subgroup analyses, meta-regression analyses*, alternative network geometries studied, alternative choice of prior distributions for Bayesian analyses,* and so forth).	**SM25-33**
**DISCUSSION**			
Summary of evidence	24	Summarize the main findings, including the strength of evidence for each main outcome; consider their relevance to key groups (e.g., healthcare providers, users, and policy-makers).	**15-17**
Limitations	25	Discuss limitations at study and outcome level (e.g., risk of bias), and at review level (e.g., incomplete retrieval of identified research, reporting bias). *Comment on the validity of the assumptions, such as transitivity and consistency. Comment on any concerns regarding network geometry (e.g.,*	**17**

		avoidance of certain comparisons).	
Conclusions	26	Provide a general interpretation of the results in the context of other evidence, and implications for future research.	**17**
**FUNDING**			
Funding	27	Describe sources of funding for the systematic review and other support (e.g., supply of data); role of funders for the systematic review. This should also include information regarding whether funding has been received from manufacturers of treatments in the network and/or whether some of the authors are content experts with professional conflicts of interest that could affect use of treatments in the network.	**17-18**

PICOS = population, intervention, comparators, outcomes, study design.

* Text in italics indicateS wording specific to reporting of network meta-analyses that has been added to guidance from the PRISMA statement.

Figure S1 The Search Strategies of Pubmed

Number	Search terms
1	exp Knee osteoarthritis/Osteoarthritis, knee
2	exp knee joint osteoarthritis/KOA
3	Osteoarthritis of knee .ti,ab
4	Knee osteoarthritides .ti,ab
5	or 1-4
7	Non-pharmacological Intervention .ti,ab
8	Non-drug therapy .ti,ab
9	Extracorporeal shock wave .ti,ab
10	Radiofrequency .ti,ab
11	Acupotomy .ti,ab
12	Laser therapy .ti,ab
13	Tuina .ti,ab
14	Kinesio taping .ti,ab
15	Electroacupuncture .ti,ab
16	Platelet-rich plasma .ti,ab
17	Ozone therapy .ti,ab
18	or 7-17
19	exp Randomized controlled clinical trial .pt
20	exp Controlled clinical trial .pt
21	Randomised .ti,ab
22	Randomized .ti,ab
23	Randomly .ti,ab
24	Trial .ti,ab
25	Groups.ti,ab
26	or 19-25
27	exp Animals/not humans.sh.
28	26 not 27
29	5 and 18 and 28

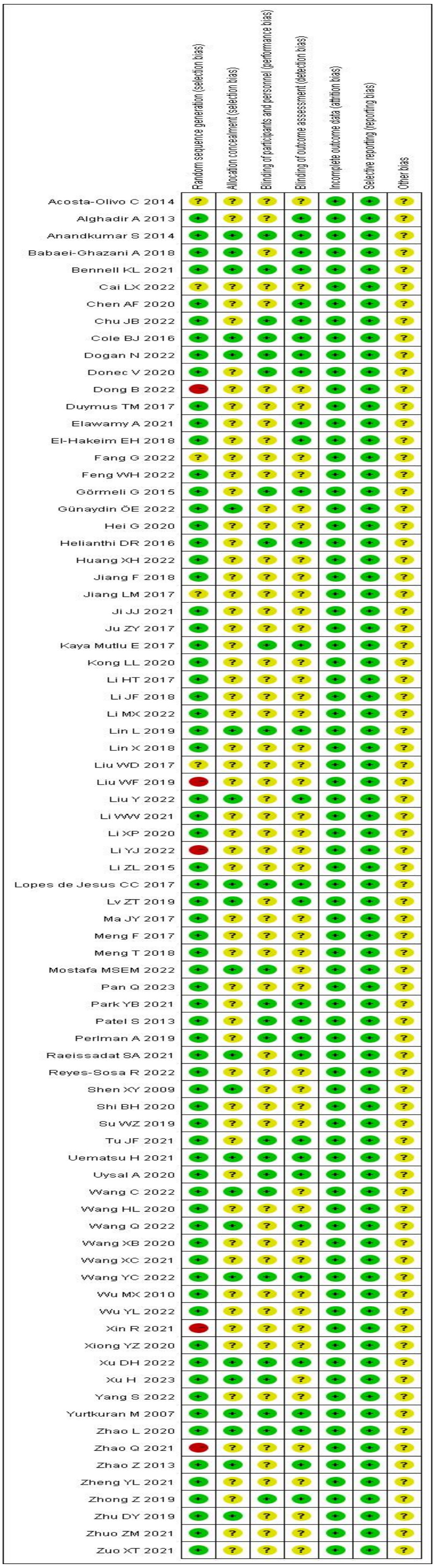

Figure S2 Risk of bias summary

Node-splitting test

Table S2 Node-splitting test of VAS (short-term)

Side	Direct		Indirect		Difference		P
	Coef.	Std. Err.	Coef.	Std. Err.	Coef.	Std. Err.	
SI VS NOD	-0.7048	0.5923	-0.7970	0.2000	0.0922	0.6248	0.883
SI VS ESW	-1.4228	0.3276	-1.8376	0.2584	0.4148	0.4172	0.320
SI VS RF	-1.7400	0.6626	-1.4743	0.2648	-0.2656	0.7135	0.710
SI VS AT	-1.4700	0.5435	-1.1910	0.3875	-0.2789	0.6675	0.676
SI VS LT	-0.7661	0.2704	-0.7283	0.4603	-0.0378	0.5335	0.943
SI VS TT	-1.3555	0.3897	-1.5967	0.3664	0.2412	0.5350	0.652
SI VS KT	-0.6807	0.3184	-0.8235	0.4516	0.1428	0.5526	0.796
SI VS EA	-1.1800	0.5253	-1.5443	0.2764	0.3643	0.5936	0.539
SI VS PRP	-1.5986	0.2879	-1.4194	0.2706	-0.1792	0.3952	0.650
SI VS ESW	-1.0915	0.3139	-0.7391	0.3244	-0.3523	0.4515	0.435
NSAIDs VS RF	-0.6355	0.3665	-0.8527	0.3375	0.2172	0.4982	0.663
NSAIDs VS TT	-0.5075	0.4125	-0.8888	0.3563	0.3812	0.5450	0.484
NSAIDs VS EA	-1.2873	0.3760	-0.3500	0.2996	-0.9373	0.4802	0.051
NSAIDs VS PRP	-0.7000	0.7164	-0.7517	0.2604	0.0517	0.7623	0.946
NOD VS ESW	-0.9503	0.3896	-0.8658	0.2551	-0.0844	0.4657	0.856
NOD VS RF	-0.9264	0.3044	-0.5321	0.2953	-0.3943	0.4241	0.353
NOD VS AT	-0.5999	0.5658	-0.4506	0.3816	-0.1493	0.6825	0.827
NOD VS LT	0.3000	0.5488	-0.0560	0.3131	0.3560	0.6318	0.573
NOD VS KT	0.2100	0.5252	-0.0047	0.3430	0.2147	0.6273	0.732
NOD VS EA	-0.9437	0.3923	-0.5156	0.3070	-0.4280	0.4981	0.390
NOD VS PRP	-0.7042	0.2145	-0.7365	0.2871	0.0323	0.3590	0.928
ESW VS LT	0.5799	0.6697	0.9975	0.3126	-0.4175	0.7391	0.572
ESW VS KT	0.4799	0.6778	1.0646	0.3335	-0.5846	0.7554	0.439
ESW VS PRP	0.7199	0.5143	0.0452	0.2512	0.6746	0.5724	0.239
RF VS PRP	0.1300	0.5257	-0.0245	0.2692	0.1545	0.5907	0.794
RF VS OT	-0.0300	0.6083	1.0571	0.3084	-1.0871	0.6820	0.111
AT VS EA	0.5800	0.5659	-0.5441	0.3922	1.1241	0.6885	0.103
TT VS EA	0.3598	0.3973	-0.2755	0.3672	0.6353	0.5409	0.240
PRP VS OT	0.9646	0.4188	0.7384	0.3211	0.2262	0.5262	0.667

Table S3 Node-splitting test of VAS (long-term)

Side	Direct		Indirect		Difference		P
	Coef.	Std. Err.	Coef.	Std. Err.	Coef.	Std. Err.	
SI VS NOD	-0.8599	0.6489	-0.3137	0.2828	-0.5462	0.7079	0.440
SI VS ESW	-1.5226	0.2640	-1.6713	0.7548	0.1486	0.7994	0.852
SI VS RF	-1.5500	0.6731	-1.4735	0.3399	-0.0764	0.7540	0.919
SI VS AT	-1.0600	0.6210	-1.6275	0.4752	0.5675	0.7820	0.468
SI VS TT	-1.2000	0.6757	-0.8890	0.5605	-0.3109	0.8779	0.723
NSAIDs VS ESW	-1.5099	0.6567	-1.3611	0.4560	-0.1488	0.7995	0.852
NSAIDs VS RF	-2.1000	0.5816	-1.0023	0.4058	-1.0976	0.7092	0.122
NSAIDs VS AT	-0.6999	0.6853	-1.5936	0.4907	0.8936	0.8429	0.289
NSAIDs VS TT	-0.8099	0.6247	-0.9579	0.6021	0.1479	0.8676	0.865
NSAIDs VS PRP	-0.9942	0.4720	-1.3282	0.4097	0.3340	0.62505	0.593
NOD VS RF	-0.9824	0.3025	-1.2433	0.3701	0.2608	0.4780	0.585
NOD VS AT	-1.5999	0.6429	-0.7297	0.4517	-0.8702	0.7857	0.268
NOD VS EA	-0.8800	0.6586	-0.4443	0.5758	-0.4356	0.8748	0.618
NOD VS PRP	-0.9312	0.2439	-0.8759	0.3283	-0.0553	0.4092	0.892
NOD VS OT	-0.1348	0.2465	-0.1758	0.4394	0.0409	0.5034	0.935
RF VS PRP	0.2599	0.6256	0.1566	0.2936	0.1033	0.6911	0.881
RF VS OT	0.4100	0.6492	1.0742	0.3207	-0.6642	0.7241	0.359
AT VS EA	0.6999	0.6380	0.0758	0.6292	0.6241	0.8961	0.486
TT VS EA	-0.1000	0.6296	0.0802	0.6987	-0.1802	0.9406	0.848
PRP VS OT	1.4694	0.4395	0.4492	0.3006	1.0201	0.5322	0.055

Table S4 Node-splitting test of WOMAC pain (short-term)

Side	Direct		Indirect		Difference		P
	Coef.	Std. Err.	Coef.	Std. Err.	Coef.	Std. Err.	
SI VS ESW	-3.7716	1.0686	-7.0129	1.5189	3.2413	1.8570	0.081
SI VS RF	-3.0200	1.8786	-3.2230	1.1134	0.2030	2.1838	0.926
SI VS ESW	-5.1000	1.7871	-4.2150	1.1849	-0.8849	2.1442	0.680
SI VS TT	-1.0700	1.7587	-2.5781	1.1978	1.5081	2.1279	0.478
SI VS PRP	-3.1707	1.2875	-1.4937	1.1867	-1.6769	1.7518	0.338
SI VS OT	-3.8099	1.7734	-0.7434	1.4018	-3.0665	2.2605	0.175
NSAIDs VS RF	-4.1601	1.7419	-6.3146	2.0446	2.1545	2.6860	0.422
NSAIDs VS TT	-3.7135	1.3984	-1.9595	1.4075	-1.7540	1.9837	0.377
NSAIDs VS EA	-0.3199	1.9604	-0.5196	1.2675	0.1996	2.3345	0.932
NOD VS RF	-6.1099	1.7360	-3.8879	2.0472	-2.2220	2.6842	0.408
NOD VS AT	-2.9094	1.2801	-0.5068	1.2061	-2.4025	1.7600	0.172
NOD VS KT	-5.6600	2.2801	-2.1464	1.1196	-3.5135	2.3462	0.134
NOD VS EA	-1.8530	3.2801	0.1802	1.1954	-2.0332	1.9655	0.301
ESW VS RF	2.3900	6.2801	1.2234	1.3986	1.1666	2.2420	0.603
ESW VS TT	2.8999	7.2801	-0.8552	1.3411	3.7551	2.3519	0.110
RF VS EA	3.7700	8.2801	-0.0327	1.1700	3.8027	2.1758	0.081
TT VS EA	0.5700	9.2801	3.3581	1.2496	-2.7880	2.1097	0.186
PRP VS OT	0.1518	10.2801	0.6946	1.7580	-0.5428	2.1830	0.804

Table S5 Node-splitting test of WOMAC pain (long-term)

Side	Direct		Indirect		Difference		P
	Coef.	Std. Err.	Coef.	Std. Err.	Coef.	Std. Err.	
SI VS AT	-7.9499	2.4551	-3.0975	1.8635	-4.8524	3.0823	0.115
SI VS TT	-4.0599	2.6087	-3.9814	1.9190	-0.0785	3.2385	0.981
SI VS EA	-1.1500	2.4047	-6.7572	2.4115	5.6072	3.4056	0.100
SI VS PRP	-2.8576	1.7818	-3.8818	1.6666	1.0242	2.4406	0.675
SI VS OT	-3.9800	2.5148	-1.9754	1.8181	-2.0045	3.1033	0.518
NSAIDs VS AT	-1.1299	2.4318	-6.0359	2.0715	4.9059	3.1944	0.125
NSAIDs VS TT	-3.3061	1.8809	-2.9218	2.0950	-0.3843	2.8151	0.891
NOD VS AT	-1.4299	2.5225	-4.2010	1.8568	2.7710	3.1322	0.376
NOD VS TT	-2.1822	1.8474	-2.5682	1.9272	0.38598	2.6697	0.885
NOD VS OT	-0.3315	1.4582	-2.6689	2.2573	2.3373	2.6814	0.383
AT VS EA	2.7999	2.6595	-0.8112	2.5209	3.6112	3.6644	0.324
PRP VS OT	0.7196	1.4717	0.7958	2.2133	-0.0762	2.6507	0.977

Table S6 Node-splitting test of WOMAC function (short-term)

Side	Direct		Indirect		Difference		P
	Coef.	Std. Err.	Coef.	Std. Err.	Coef.	Std. Err.	
SI VS NOD	-2.8653	4.5890	-3.0856	1.9859	0.2202	4.9889	0.965
SI VS ESW	-5.5578	2.1300	-11.9897	3.1602	6.4319	3.8110	0.091
SI VS RF	-11.8000	3.7140	-9.0684	2.2448	-2.7315	4.3397	0.529
SI VS TT	-9.2999	3.7206	-13.3340	2.4547	4.0340	4.4574	0.365
SI VS EA	-2.5200	3.6825	-3.5595	2.6071	1.0395	4.5120	0.818
SI VS PRP	-9.5923	2.6233	-5.5230	2.5041	-4.0692	3.6271	0.262
SI VS OT	-16.9081	4.0406	-7.2623	2.9099	-9.6457	5.0410	0.056
NSAIDs VS RF	-9.6700	3.5833	-15.0172	4.2824	5.3471	5.5838	0.338
NSAIDs VS AT	-13.7000	3.6050	-12.3235	2.8043	-1.3764	4.5673	0.763
NSAIDs VS TT	-14.3336	3.0351	-16.0170	3.0841	1.6833	4.2989	0.695
NSAIDs VS EA	-12.0000	6.5714	-5.3096	2.5834	-6.6903	7.0609	0.343
NSAIDs VS PRP	-11.3199	3.6857	-10.0260	2.8221	-1.2939	4.6421	0.780
NOD VS RF	-8.0399	3.6026	-2.6826	4.2650	-5.3572	5.5829	0.337
NOD VS AT	-6.3801	2.4720	-7.1372	2.5598	0.7571	3.5592	0.832
NOD VS TT	-11.1299	3.8088	-8.2227	2.4336	-2.9072	4.5199	0.520
NOD VS EA	-3.0383	2.9074	2.1203	2.5664	-5.1587	3.8792	0.184
NOD VS OT	-7.7582	2.8009	-7.0758	3.8430	-0.6824	4.7770	0.886
ESW VS RF	0.7998	3.4744	-4.1700	2.7673	4.9699	4.4418	0.263
ESW VS TT	-1.3998	4.3730	-5.8758	2.8486	4.4760	5.2190	0.391
AT VS EA	7.9900	4.1100	6.0392	2.5692	1.9508	4.8469	0.687
TT VS EA	14.2899	3.9512	6.5176	2.6133	7.7723	4.7372	0.101
PRP VS OT	-0.5178	2.6229	-7.7346	3.4879	7.2168	4.3566	0.098

Table S7 Node-splitting test of WOMAC function (long-term)

Side	Direct		Indirect		Difference		P
	Coef.	Std. Err.	Coef.	Std. Err.	Coef.	Std. Err.	
SI VS TT	-11.6600	4.0351	-14.4254	3.0258	2.7654	5.0435	0.583
SI VS PRP	-6.4470	2.6729	-6.6663	2.5557	0.2193	3.6973	0.953
SI VS OT	-4.9699	3.7731	-4.8752	2.8031	-0.0947	4.7004	0.984
NSAIDs VS RF	-13.5301	3.4106	-6.4738	3.2373	-7.0562	4.7024	0.133
NSAIDs VS AT	-4.8300	3.4589	-11.3198	2.9084	6.4898	4.5192	0.151
NSAIDs VS TT	-10.3804	3.0673	-12.2358	3.2088	1.8554	4.4307	0.675
NSAIDs VS PRP	-5.1536	2.6472	-3.5904	2.7300	-1.5631	3.8039	0.681
NOD VS RF	-6.1527	2.4958	-13.1868	3.9845	7.0340	4.7016	0.135
NOD VS AT	-6.2423	3.1181	-7.4834	2.7203	1.2411	4.1391	0.764
NOD VS TT	-11.4716	3.0142	-7.6384	2.9319	-3.8332	4.2064	0.362
NOD VS EA	-8.9699	4.3156	-1.9934	3.0278	-6.9765	5.2718	0.186
NOD VS PRP	-2.4586	1.5280	-3.4115	2.8969	0.9528	3.2755	0.771
NOD VS OT	-1.4656	2.3286	0.0441	3.5642	-1.5097	4.2516	0.723
RF VS EA	1.0099	3.5998	4.1553	3.5058	-3.1453	5.0249	0.531
PRP VS OT	2.0996	2.2762	0.5909	3.4762	1.5086	4.1237	0.714

Table S8 Node-splitting test of ROM (short-term)

Side	Direct		Indirect		Difference		P
	Coef.	Std. Err.	Coef.	Std. Err.	Coef.	Std. Err.	
SI VS ESW	8.4000	6.7785	11.2671	6.3704	-2.8671	9.3022	0.758
SI VS AT	7.9000	6.0835	24.0128	9.9843	-16.1128	11.6917	0.168
SI VS TT	12.8000	6.7416	15.8907	5.4835	-3.0900	8.6901	0.722
SI VS KT	2.7021	3.8473	12.8080	9.0353	-10.1058	9.8210	0.303
SI VS EA	12.5600	6.1444	-0.9322	6.2489	13.4922	8.7637	0.124
NSAIDs VS AT	15.7900	6.7121	-0.3304	9.5753	16.1204	11.6936	0.168
NSAIDs VS TT	8.4500	6.1100	24.5786	9.9693	-16.1286	11.6927	0.168
NOD VS LT	4.0999	6.8727	-3.7397	654.650	7.8397	654.689	0.990
NOD VS KT	6.4499	6.2269	-3.6758	7.6044	10.1258	9.8286	0.303
NOD VS PRP	8.4772	4.9226	4.7805	9.5893	3.6967	10.7792	0.732
ESW VS TT	4.5997	7.2928	4.8763	7.3165	-0.2765	10.3304	0.979
ESW VS PRP	-1.0259	4.6621	2.5972	9.7231	-3.6232	10.7830	0.737
TT VS EA	-12.1122	4.4056	1.3780	7.5767	-13.4903	8.7641	0.124

Table S9 Node-splitting test of IL-1β

Side	Direct		Indirect		Difference		P
	Coef.	Std. Err.	Coef.	Std. Err.	Coef.	Std. Err.	
SI VS TT	-7.7000	7.8836	-15.2132	10.2505	7.5132	12.9316	0.561
SI VS PRP	-15.7900	7.0052	-6.1027	7.7087	-9.6872	10.4162	0.352
NSAIDs VS ESW	-11.8297	7.2850	-11.9308	7.0487	0.1011	10.1369	0.992
NSAIDs VS AT	3.2050	4.7725	-10.0074	8.1076	13.2125	9.4079	0.160
NSAIDs VS TT	-10.9600	7.5136	-3.4391	10.5273	-7.5208	12.9337	0.561
NSAIDs VS EA	-3.4610	5.3431	2.6597	8.9438	-6.1208	10.4185	0.557
NSAIDs VS PRP	-10.9199	7.4242	-8.2699	5.9680	-2.6500	9.5255	0.781
NOD VS ESW	-15.5279	5.1496	-14.8878	8.6647	-0.6400	10.0794	0.949
NOD VS RF	-22.9809	4.9929	-2.8575	134.035	-20.1233	134.129	0.881
NOD VS AT	-10.4900	6.7462	2.7529	6.5535	-13.2429	9.4054	0.159
NOD VS EA	-2.0700	7.6081	-8.1839	7.1176	6.1138	10.4184	0.557
NOD VS PRP	-10.7501	4.1001	-18.4914	6.8872	7.7412	8.0153	0.334
NOD VS OT	-8.0532	4.1970	-0.4613	23.0734	-7.5918	23.4522	0.746

Table S10 Node-splitting test of TNF-α

Side	Direct		Indirect		Difference		P
	Coef.	Std. Err.	Coef.	Std. Err.	Coef.	Std. Err.	
SI VS TT	-14.1000	6.6466	-12.8651	9.7095	-1.2348	11.7665	0.916
SI VS PRP	-13.1000	6.3388	-9.8183	8.7048	-3.2816	10.7682	0.761
NSAIDs VS ESW	-12.0076	3.7680	-10.1410	7.2348	-1.8665	8.1584	0.819
NSAIDs VS RF	-8.7800	6.4601	-12.3471	5.6855	3.5670	8.6057	0.679
NSAIDs VS TT	-7.1000	6.8742	-8.3288	9.5521	1.2288	11.7685	0.917
NSAIDs VS EA	-9.9939	4.7851	-2.2204	5.4933	-7.7735	7.2648	0.285
NSAIDs VS PRP	-11.7498	6.1905	-2.6970	4.4742	-9.0528	7.6381	0.236
NOD VS ESW	-12.5000	6.3764	-14.1153	5.1076	1.6153	8.1698	0.843
NOD VS RF	-13.6750	4.5780	-10.0748	7.2842	-3.6002	8.6032	0.676
NOD VS EA	-5.7010	4.3288	-13.4506	5.8347	7.7495	7.2652	0.286
NOD VS PRP	-5.3125	3.5510	-14.3768	6.1230	9.0642	7.0773	0.200
NOD VS OT	-4.6322	3.6678	10.1887	36.5228	-14.8209	36.7085	0.686

Evidence network diagram

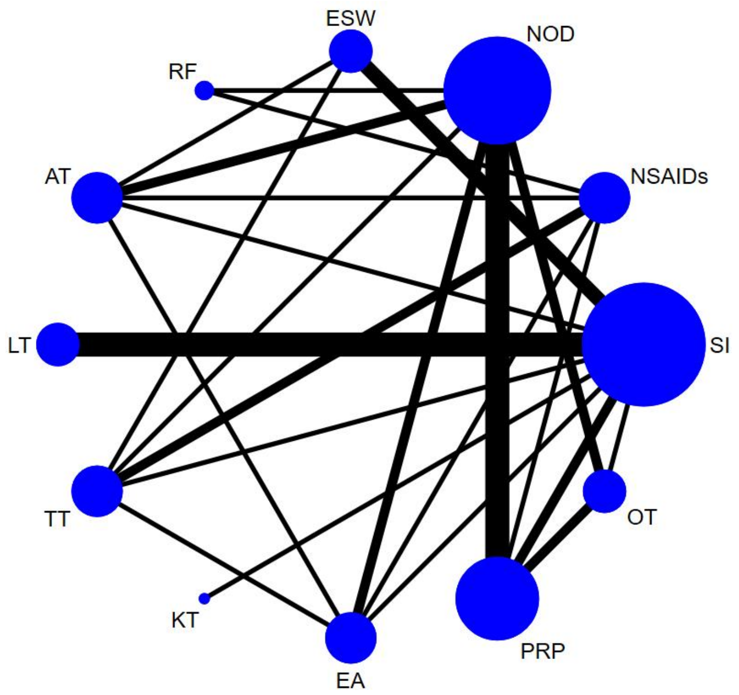

Figure S3 Network diagram of WOMAC pain (short-term)

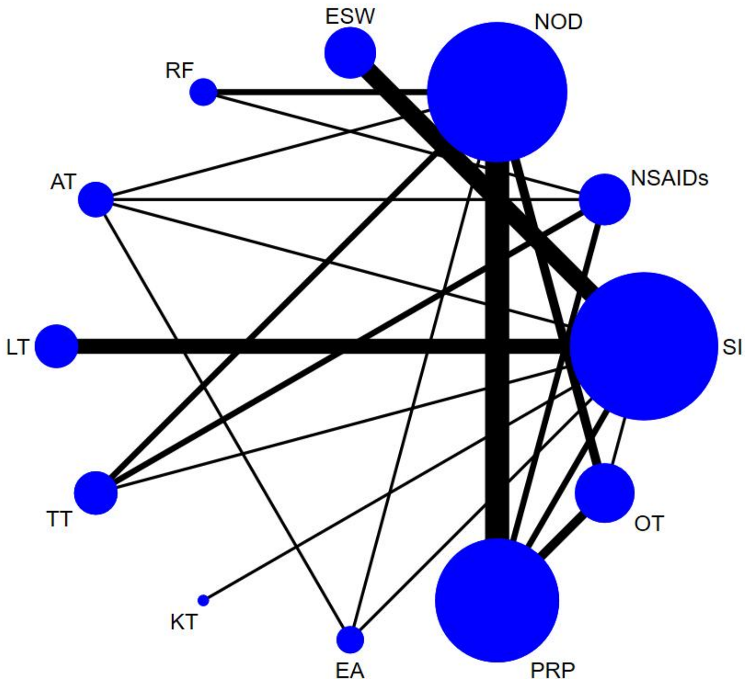

Figure S4 Network diagram of WOMAC pain (long-term)

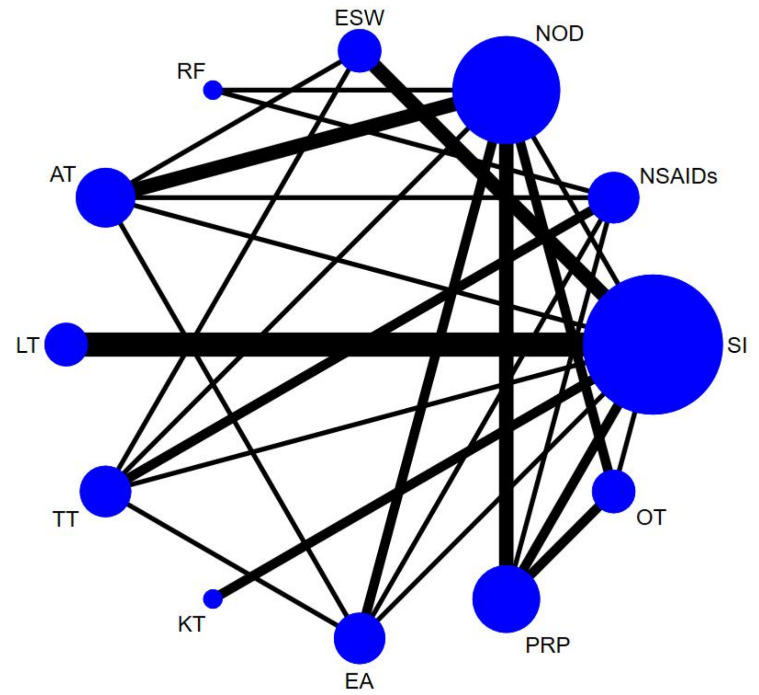

Figure S5 Network diagram of WOMAC function (short-term)

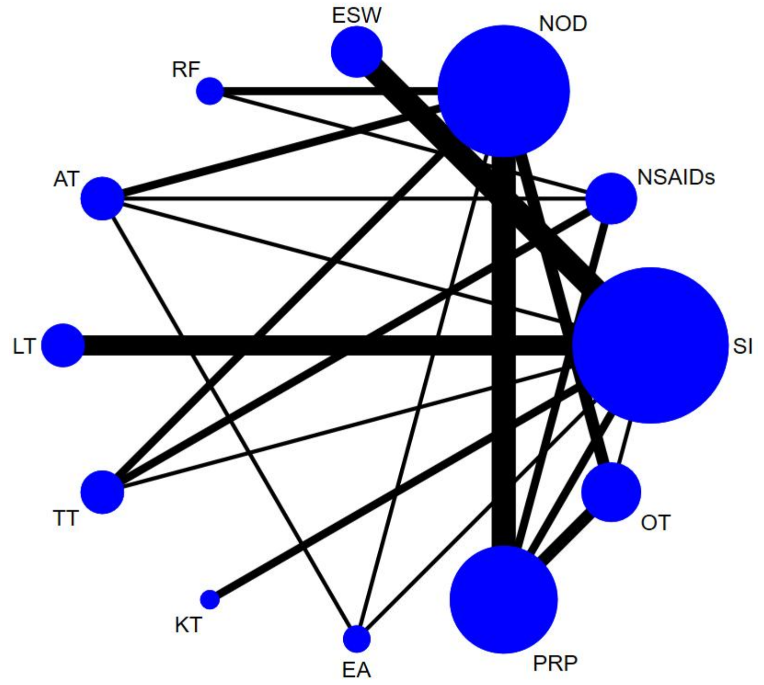

Figure S6 Network diagram of WOMAC function (long-term)

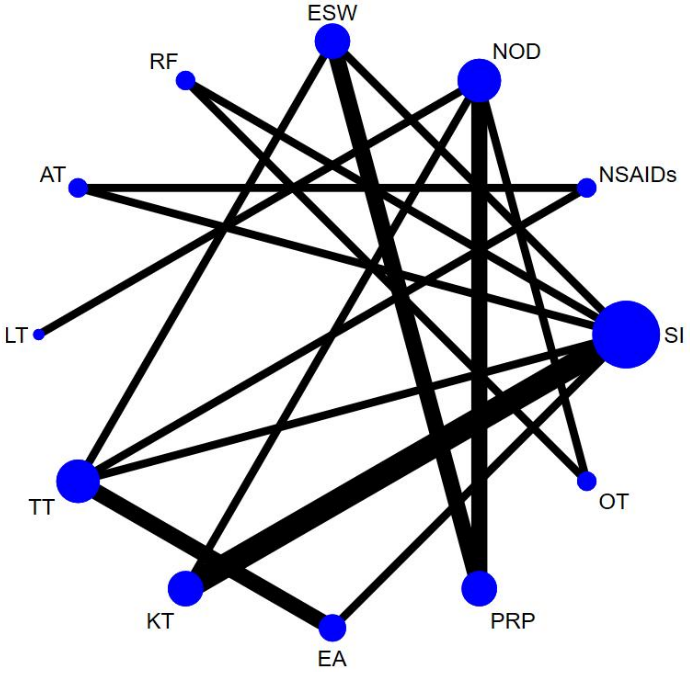

Figure S7 Network diagram of ROM (short-term)

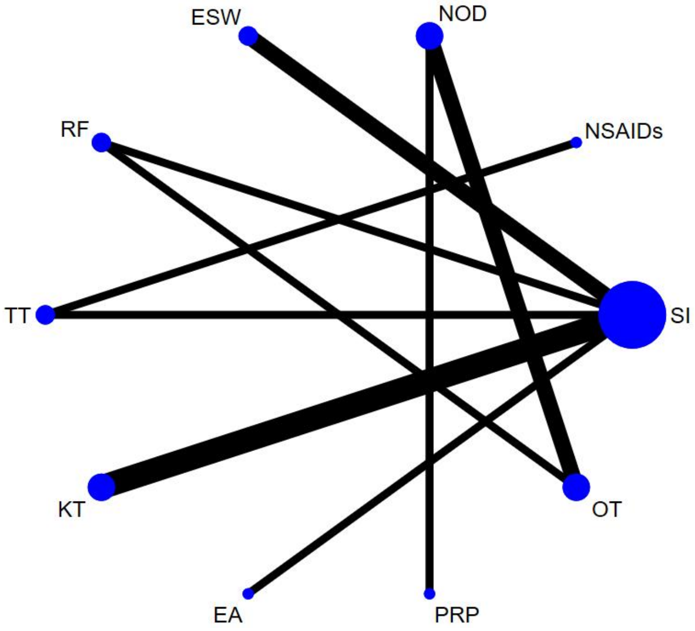

Figure S8 Network diagram of ROM (long-term)

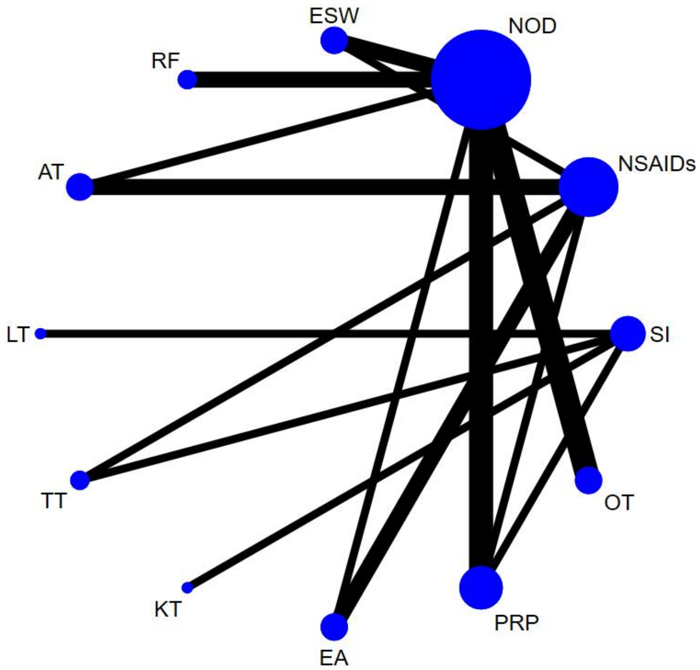

Figure S9 Network diagram of IL-1β

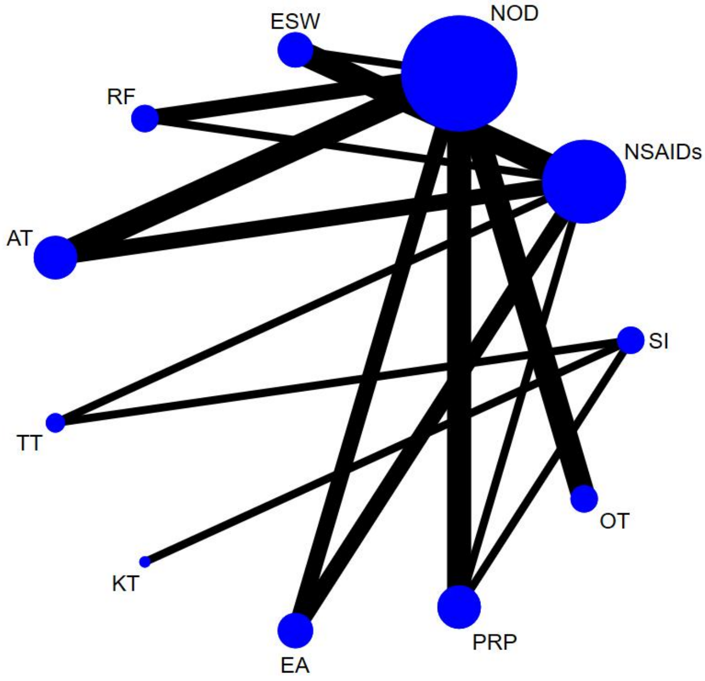

Figure S10 Network diagram of TNF-α

The results of network meta-analysis

	WOMAC pain (Long-term)											
WOMAC pain (Short-term)	**RF**	-1.48 (-5.38,2.42)	-2.34 (-5.96,1.28)	-1.74 (-6.00,2.52)	-1.48 (-5.38,2.42)	-2.95 (-6.09,0.20)	-2.38 (-6.84,2.08)	**-3.69 (-7.36,-0.02)**	**-5.47 (-8.70,-2.25)**	-4.16 (-10.28,1.97)	**-4.70 (-7.60,-1.81)**	**-6.36 (-10.00,-2.71)**
	-1.86 (-5.04,1.32)	**ESW**	-0.83 (-4.42,2.76)	-0.23 (-3.22,2.76)	-0.03 (-3.63,3.57)	-1.43 (-4.53,1.66)	-0.87 (-4.81,3.08)	-2.17 (-5.66,1.31)	**-3.96 (-7.55,-0.36)**	-2.64 (-7.95,2.67)	**-3.19 (-6.36,-0.02)**	**-4.84 (-6.85,-2.84)**
	-2.23 (-5.16,0.71)	-0.36 (-2.56,1.84)	**TT**	-0.60 (-4.31,3.11)	-0.86 (-4.33,2.61)	-0.61 (-3.33,2.12)	-0.04 (-4.10,4.02)	-1.35 (-4.65,1.95)	**-3.13 (-5.83,-0.43)**	-1.81 (-7.56,3.94)	-2.36 (-4.93,0.21)	**-4.01 (-6.99,-1.03)**
	-3.17 (-6.50,0.17)	-1.30 (-3.72,1.11)	-0.94 (-3.46,1.58)	**LT**	-0.26 (-3.99,3.46)	-1.20 (-4.44,2.03)	-0.64 (-4.70,3.43)	-1.94 (-5.55,1.66)	**-3.73 (-7.44,-0.01)**	-2.41 (-7.81,2.98)	-2.96 (-6.27,0.35)	**-4.61 (-6.83,-2.40)**
	**-3.54 (-6.44,-0.64)**	-1.68 (-3.79,0.44)	-1.31 (-3.44,0.82)	-0.37 (-2.84,2.10)	**AT**	-1.47 (-4.47,1.54)	-0.90 (-4.45,2.65)	-2.21 (-5.71,1.30)	**-3.99 (-7.13,-0.85)**	-2.68 (-8.43,3.08)	**-3.22 (-6.13,-0.32)**	**-4.88 (-7.87,-1.88)**
	**-4.45 (-7.22,-1.67)**	**-2.58 (-4.83,-0.34)**	**-2.22 (-4.30,-0.13)**	-1.28 (-3.64,1.09)	-0.91 (-2.88,1.06)	**PRP**	-0.57 (-4.18,3.05)	-0.74 (-3.10,1.62)	**-2.52 (-4.98,-0.07)**	-1.21 (-6.66,4.24)	**-1.76 (-3.31,-0.21)**	**-3.41 (-5.77,-1.05)**
	**-4.61 (-7.54,-1.67)**	**-2.75 (-5.09,-0.40)**	**-2.38 (-4.39,-0.38)**	-1.44 (-3.97,1.09)	-1.07 (-3.07,0.93)	-0.16 (-2.21,1.88)	**EA**	-1.31 (-5.32,2.70)	-3.09 (-7.05,0.87)	-1.78 (-7.76,4.20)	-3.09 (-7.05,0.87)	**-3.98 (-7.38,-0.58)**
	**-4.79 (-7.98,-1.60)**	**-2.93 (-5.59,-0.26)**	-2.56 (-5.14,0.01)	-1.62 (-4.36,1.12)	-1.25 (-3.73,1.22)	-0.34 (-2.38,1.69)	-0.18 (-2.72,2.36)	**OT**	-1.78 (-4.97,1.41)	-0.47 (-6.15,5.22)	-1.02 (-3.40,1.37)	-2.67 (-5.52,0.18)
	**-5.07 (-7.65,-2.48)**	**-3.20 (-5.62,-0.79)**	**-2.84 (-4.78,-0.90)**	-1.90 (-4.54,0.74)	-1.53 (-3.57,0.51)	-0.62 (-2.62,1.38)	-0.46 (-2.51,1.59)	-0.28 (-2.85,2.30)	**NSAIDs**	-1.32 (-7.07,4.44)	-0.77 (-3.26,1.72)	-0.88 (-3.87,2.10)
	**-5.51 (-10.14,-0.88)**	-3.65 (-7.67,0.37)	-3.28 (-7.37,0.80)	-2.34 (-6.31,1.63)	-1.97 (-6.02,2.08)	-1.06 (-5.05,2.92)	-0.90 (-4.99,3.19)	-0.72 (-4.94,3.50)	-0.44 (-4.60,3.72)	**KT**	-0.55 (-6.05,4.95)	-2.20 (-7.12,2.72)
	**-5.18 (-7.76,-2.60)**	**-3.32 (-5.52,-1.11)**	**-2.95 (-4.92,-0.99)**	-2.01 (-4.41,0.38)	-1.64 (-3.41,0.12)	-0.74 (-2.10,0.63)	-0.57 (-2.43,1.29)	-0.39 (-2.44,1.65)	-0.11 (-2.05,1.82)	-0.33 (-3.68,4.34)	**NOD**	-1.65 (-4.11,0.81)
	**-6.71 (-9.61,-3.81)**	**-4.85 (-6.61,-3.08)**	**-4.48 (-6.39,-2.58)**	**-3.54 (-5.19,-1.89)**	**-3.17 (-5.01,-1.33)**	**-2.26 (-3.96,-0.57)**	**-2.10 (-4.02,-0.18)**	-1.92 (-4.11,0.27)	-1.64 (-3.71,0.42)	-1.20 (-4.81,2.41)	-1.53 (-3.27,0.21)	**SI**

Figure S11 Network meta-analysis of WOMAC pain in short- and long-term [SMD(95% CI)]

	WOMAC function (Long-term)											
WOMAC function (Short-term)	**TT**	-1.38 (-7.01,4.25)	-2.55 (-7.69,2.59)	**-7.32 (-13.05,-1.59)**	-1.38 (-7.01,4.25)	-4.12 (-9.71,1.47)	**-6.83 (-11.25,-2.42)**	**-9.29 (-16.47,-2.11)**	-5.19 (-11.14,0.76)	**-9.50 (-13.68,-5.31)**	**-13.41 (-18.07,-8.74)**	**-11.21 (-15.55,-6.87)**
	-1.53 (-6.98,3.93)	**OT**	**-5.92 (-10.96,-0.89)**	-1.15 (-6.63,4.32)	-5.94 (-12.35,0.46)	-4.36 (-9.67,0.96)	-1.64 (-5.33,2.05)	-0.82 (-7.80,6.16)	-3.28 (-9.06,2.49)	-1.02 (-4.79,2.75)	**-4.93 (-9.26,-0.61)**	-2.73 (-7.57,2.11)
	-2.30 (-6.67,2.07)	-0.77 (-5.91,4.37)	**AT**	-4.77 (-10.18,0.65)	-1.17 (-6.71,4.37)	-1.57 (-6.83,3.69)	**-4.28 (-8.49,-0.07)**	-6.74 (-13.67,0.19)	-2.64 (-7.52,2.25)	**-6.95 (-10.89,-3.00)**	**-10.85 (-15.11,-6.60)**	**-8.66 (-13.09,-4.22)**
	-2.58 (-7.87,2.70)	-1.06 (-6.97,4.86)	-0.29 (-5.37,4.80)	**LT**	-5.94 (-12.35,0.46)	-3.20 (-7.76,1.36)	-0.49 (-5.37,4.40)	-1.97 (-8.38,4.44)	-2.13 (-8.02,3.76)	-2.18 (-7.20,2.84)	**-6.09 (-9.43,-2.74)**	-3.89 (-9.49,1.71)
	-3.24 (-9.32,2.84)	-1.71 (-8.38,4.96)	-0.94 (-6.86,4.97)	-0.65 (-7.60,6.29)	**RF**	-2.74 (-9.01,3.53)	**-5.45 (-10.23,-0.68)**	**-7.91 (-15.64,-0.18)**	-3.81 (-10.18,2.56)	**-8.12 (-12.43,-3.81)**	**-12.03 (-17.48,-6.57)**	**-9.83 (-14.61,-5.05)**
	-4.54 (-9.16,0.08)	-3.01 (-8.67,2.64)	-2.24 (-6.54,2.06)	-1.96 (-6.96,3.04)	-1.30 (-7.87,5.27)	**ESW**	-2.71 (-7.42,2.00)	-5.17 (-11.46,1.11)	-1.07 (-6.82,4.68)	**-5.38 (-10.23,-0.53)**	**-9.29 (-12.38,-6.19)**	**-7.09 (-12.53,-1.65)**
	**-4.64 (-9.07,-0.22)**	-3.12 (-7.36,1.12)	-2.35 (-6.44,1.75)	-2.06 (-7.05,2.93)	-1.41 (-7.27,4.46)	-0.10 (-4.80,4.59)	**PRP**	-2.46 (-8.98,4.07)	-1.64 (-6.77,3.48)	**-2.67 (-5.27,-0.06)**	**-6.57 (-10.12,-3.02)**	**-4.38 (-8.06,-0.69)**
	**-8.75 (-15.46,-2.03)**	-7.22 (-14.43,-0.00)	-6.45 (-13.00,0.10)	-6.16 (-12.58,0.26)	-5.51 (-13.59,2.57)	-4.21 (-10.70,2.28)	-4.10 (-10.59,2.38)	**KT**	-4.10 (-11.41,3.21)	-0.21 (-6.83,6.42)	-4.11 (-9.59,1.36)	-1.92 (-8.99,5.16)
	**-8.90 (-13.31,-4.49)**	**-7.37 (-12.82,-1.93)**	**-6.60 (-10.80,-2.41)**	**-6.32 (-11.68,-0.96)**	-5.66 (-11.89,0.57)	-4.36 (-9.31,0.59)	-4.26 (-8.73,0.22)	-0.15 (-6.93,6.62)	**EA**	-4.31 (-9.23,0.62)	**-8.22 (-13.06,-3.37)**	**-6.02 (-11.66,-0.38)**
	**-9.05 (-13.04,-5.06)**	**-7.52 (-11.85,-3.20)**	**-6.75 (-10.17,-3.34)**	**-6.47 (-11.42,-1.52)**	**-5.81 (-11.15,-0.47)**	**-4.51 (-8.98,-0.04)**	**-4.41 (-7.58,-1.23)**	-0.30 (-6.75,6.15)	-0.15 (-3.99,3.70)	**NOD**	**-3.91 (-7.64,-0.17)**	-1.71 (-5.47,2.05)
	**-12.11 (-16.09,-8.12)**	**-10.58 (-15.36,-5.79)**	**-9.81 (-13.51,-6.10)**	**-9.52 (-13.00,-6.05)**	**-8.87 (-14.88,-2.86)**	**-7.57 (-11.16,-3.97)**	**-7.46 (-11.05,-3.88)**	-3.36 (-8.76,2.04)	-3.21 (-7.29,0.88)	-3.06 (-6.58,0.47)	**SI**	-2.20 (-6.67,2.28)
	**-15.11 (-19.33,-10.89)**	**-13.58 (-19.07,-8.09)**	**-12.81 (-17.07,-8.55)**	**-12.52 (-18.17,-6.88)**	**-11.87 (-17.20,-6.53)**	**-10.57 (-15.68,-5.45)**	**-10.46 (-14.77,-6.15)**	-6.36 (-13.35,0.63)	**-6.21 (-10.97,-1.45)**	**-6.06 (-10.16,-1.96)**	-3.00 (-7.43,1.43)	**NSAIDs**

Figure S12 Network meta-analysis of WOMAC function in short- and long-term [SMD(95% CI)]

ROM (Short-term)	ROM (Long-term)											
	**TT**	-	2.96 (-1.48,7.40)	5.40 (1.13,9.66)	3.20 (-3.84,10.25)	1.24 (-4.09,6.57)	-	3.14 (-1.37,7.66)	**8.09 (3.73,12.45)**	**10.45 (7.31,13.59)**	**8.27 (2.14,14.41)**	**13.80 (10.39,17.21)**
	2.36 (-9.34,14.06)	**AT**	-	-	-	-	-	-	-	-	-	-
	3.55 (-9.49,16.59)	1.20 (-13.79,16.19)	**RF**	2.44 (-1.39,6.27)	0.24 (-5.23,5.71)	1.72 (-3.26,6.71)	-	0.18 (-0.66,1.02)	**5.13 (1.19,9.07)**	**7.49 (2.05,12.93)**	**5.31 (1.07,9.55)**	**10.84 (7.99,13.68)**
	4.74 (-4.91,14.38)	2.38 (-10.81,15.57)	1.18 (-11.71,14.08)	**ESW**	2.20 (-4.48,8.87)	4.16 (-0.67,8.99)	-	2.26 (-1.66,6.17)	2.69 (-1.05,6.43)	5.05 (-0.25,10.35)	2.87 (-2.84,8.59)	**8.40 (5.84,10.96)**
	5.09 (-6.23,16.42)	2.74 (-11.46,16.93)	1.54 (-11.63,14.71)	0.36 (-7.46,8.18)	**PRP**	1.96 (-5.43,9.36)	-	-0.06 (-5.46,5.34)	4.89 (-1.85,11.62)	7.25 (-0.47,14.96)	**5.07 (1.62,8.52)**	**10.60 (4.43,16.76)**
	**8.75 (0.84,16.67)**	6.39 (-6.65,19.44)	5.20 (-8.66,19.05)	4.02 (-7.31,15.34)	3.66 (-8.98,16.29)	**EA**	-	1.90 (-3.15,6.96)	**6.85 (1.93,11.77)**	**9.21 (3.02,15.40)**	**7.03 (0.49,13.58)**	**12.56 (8.46,16.66)**
	8.68 (-9.10,26.46)	6.32 (-13.19,25.84)	5.13 (-13.02,23.28)	3.95 (-12.73,20.62)	3.59 (-12.17,19.35)	-0.07 (-18.59,18.45)	**LT**	-	-	-	-	-
	9.13 (-4.96,23.21)	6.77 (-9.29,22.82)	5.57 (-4.98,16.12)	4.39 (-9.01,17.79)	4.03 (-9.02,17.08)	0.37 (-14.54,15.29)	0.44 (-17.10,17.98)	**OT**	**4.95 (0.92,8.97)**	**7.31 (1.80,12.81)**	**5.13 (0.97,9.29)**	**10.66 (7.69,13.62)**
	**10.40 (0.09,20.70)**	8.04 (-4.61,20.69)	6.84 (-5.25,18.93)	5.66 (-4.55,15.87)	5.30 (-5.35,15.96)	1.65 (-9.66,12.95)	1.72 (-14.77,18.21)	1.27 (-11.59,14.13)	**KT**	2.36 (-3.02,7.74)	0.18 (-5.60,5.97)	**5.71 (2.98,8.43)**
	**12.88 (2.22,23.54)**	10.52 (-0.68,21.73)	9.33 (-6.35,25.01)	8.15 (-5.31,21.60)	7.79 (-6.80,22.38)	4.13 (-8.62,16.88)	4.20 (-15.71,24.11)	3.76 (-12.86,20.37)	2.48 (-11.00,15.97)	**NSAIDs**	2.18 (-4.72,9.07)	3.35 (-1.29,7.99)
	**12.78 (1.18,24.38)**	10.42 (-3.70,24.54)	9.23 (-2.94,21.39)	8.04 (-1.79,17.88)	7.69 (-0.49,15.87)	4.03 (-8.69,16.75)	4.10 (-9.37,17.57)	3.65 (-7.58,14.89)	2.38 (-7.13,11.90)	-0.10 (-14.77,14.57)	**NOD**	**-5.53 (0.42,10.63)**
	**14.65 (6.70,22.60)**	**12.29 (1.66,22.93)**	**11.10 (0.44,21.75)**	**9.91 (1.24,18.58)**	9.56 (-0.40,19.51)	5.90 (-3.21,15.01)	5.97 (-10.60,22.53)	5.52 (-6.66,17.71)	4.25 (-2.73,11.24)	1.77 (-9.94,13.47)	1.87 (-7.77,11.51)	**SI**

Figure S13 Network meta-analysis of ROM in short- and long-term [SMD(95% CI)]

IL-1β	TNF-α											
	**RF**	-0.80 (-10.51,8.91)	-5.03 (-14.24,4.18)	-3.32 (-16.23,9.60)	-2.42 (-20.11,15.27)	-	-8.15 (-18.43,2.13)	-4.18 (-13.76,5.39)	-2.25 (-11.60,7.11)	**-10.82 (-18.98,-2.66)**	**-12.64 (-20.05,-5.23)**	**-17.04 (-29.48,-4.60)**
	-7.59 (-20.43,5.26)	**ESW**	-5.83 (-14.68,3.01)	-4.12 (-16.27,8.03)	-3.22 (-20.56,14.12)	-	-8.95 (-19.32,1.41)	-4.98 (-13.97,4.00)	-3.05 (-11.86,5.76)	**-11.62 (-17.94,-5.31)**	**-13.44 (-20.98,-5.90)**	**-17.84 (-29.77,-5.91)**
	-10.18 (-22.13,1.78)	-2.59 (-12.83,7.65)	**PRP**	-1.71 (-13.06,9.64)	-2.61 (-18.54,13.33)	-	-3.12 (-12.44,6.20)	-0.85 (-9.50,7.81)	-2.78 (-11.19,5.62)	-5.79 (-12.96,1.38)	**-7.61 (-13.69,-1.53)**	**-12.01 (-21.79,-2.23)**
	-11.07 (-27.18,5.04)	-3.48 (-17.72,10.75)	-0.89 (-13.85,12.07)	**TT**	-0.90 (-17.24,15.45)	-	-4.83 (-18.16,8.49)	-0.86 (-13.25,11.52)	-1.07 (-13.32,11.17)	-7.50 (-18.13,3.12)	-9.32 (-20.68,2.04)	**-13.72 (-24.16,-3.29)**
	-10.69 (-30.68,9.30)	-3.11 (-21.98,15.77)	-0.51 (-17.78,16.75)	0.38 (-18.08,18.84)	**KT**	-	-5.73 (-23.51,12.05)	-1.76 (-19.13,15.61)	-0.18 (-17.42,17.07)	-8.40 (-24.86,8.06)	-10.22 (-26.63,6.20)	**-14.62 (-27.20,-2.04)**
	-10.91 (-30.72,8.90)	-3.33 (-22.02,15.37)	-0.73 (-17.79,16.33)	0.16 (-18.11,18.43)	-0.22 (-19.79,19.35)	**LT**	-	-	-	-	-	-
	**-15.14 (-27.74,-2.55)**	-7.56 (-19.00,3.89)	-4.96 (-15.25,5.32)	-4.07 (-18.77,10.62)	-4.45 (-23.11,14.21)	-4.23 (-22.70,14.24)	**OT**	-3.97 (-13.86,5.92)	-5.91 (-15.56,3.75)	-2.67 (-11.93,6.59)	-4.49 (-11.62,2.64)	-8.89 (-21.45,3.67)
	**-17.61 (-31.58,-3.64)**	-10.02 (-21.86,1.81)	-7.43 (-18.56,3.70)	-6.54 (-20.82,7.74)	-6.92 (-26.25,12.41)	-6.70 (-25.85,12.45)	-2.47 (-15.11,10.18)	**EA**	-1.94 (-10.69,6.82)	-6.64 (-13.77,0.49)	**-10.39 (-16.90,-3.89)**	**-12.86 (-24.84,-0.88)**
	**-19.28 (-32.96,-5.60)**	**-11.69 (-23.20,-0.19)**	-9.10 (-19.87,1.67)	-8.21 (-22.21,5.79)	-8.59 (-27.70,10.52)	-8.37 (-27.30,10.56)	-4.14 (-16.47,8.19)	-1.67 (-13.22,9.88)	**AT**	**-8.58 (-15.54,-1.61)**	**-10.39 (-16.90,-3.89)**	**-14.80 (-26.59,-3.00)**
	**-19.47 (-32.13,-6.81)**	**-11.89 (-21.42,-2.35)**	**-9.30 (-18.11,-0.49)**	-8.40 (-20.21,3.40)	-8.78 (-26.77,9.21)	-8.56 (-26.36,9.23)	-4.33 (-15.46,6.79)	-1.86 (-10.67,6.94)	-0.19 (-8.57,8.19)	**NSAIDs**	-1.82 (-7.75,4.12)	-6.22 (-16.84,4.40)
	**-22.95 (-32.73,-13.18)**	**-15.37 (-23.70,-7.03)**	**-12.78 (-19.67,-5.88)**	-11.88 (-24.71,0.94)	-12.26 (-29.72,5.20)	-12.04 (-29.30,5.22)	-7.81 (-15.76,0.13)	-5.34 (-15.32,4.64)	-3.67 (-13.25,5.90)	-3.48 (-11.53,4.57)	**NOD**	-4.40 (-14.95,6.14)
	**-21.59 (-35.89,-7.29)**	**-14.01 (-26.71,-1.30)**	**-11.41 (-21.57,-1.26)**	-10.52 (-22.60,1.56)	-10.90 (-24.86,3.06)	-10.68 (-24.39,3.03)	-6.45 (-18.83,5.93)	-3.98 (-17.36,9.40)	-2.31 (-15.37,10.74)	-2.12 (-13.47,9.23)	-1.36 (-9.12,11.84)	**SI**

Figure S14 Network meta-analysis of IL-1β and TNF-α [SMD(95% CI)]

Funnel plot

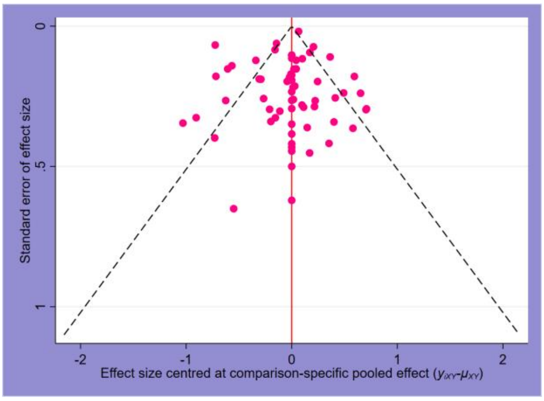

Figure S15 Funnel plot of VAS (short-term)

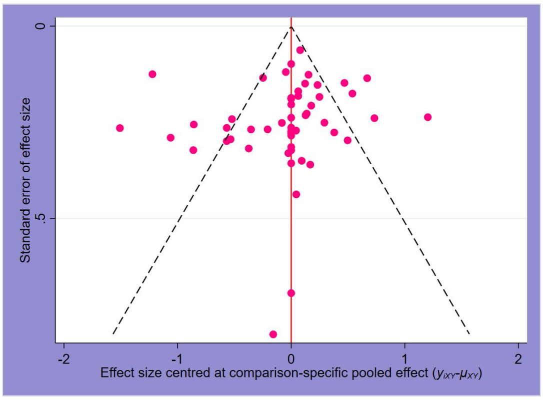

Figure S16 Funnel plot of VAS (long-term)

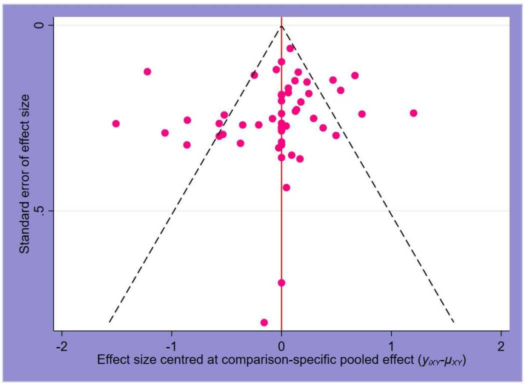

Figure S17 Funnel plot of WOMAC pain (short-term)

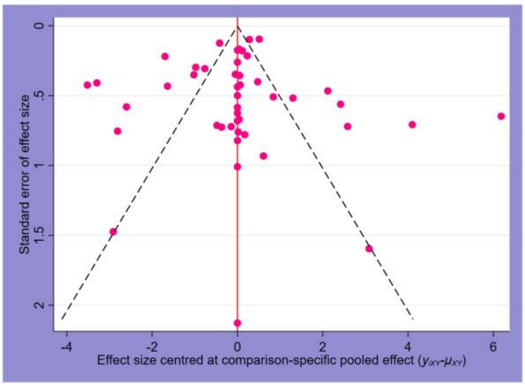

Figure S18 Funnel plot of WOMAC pain (long-term)

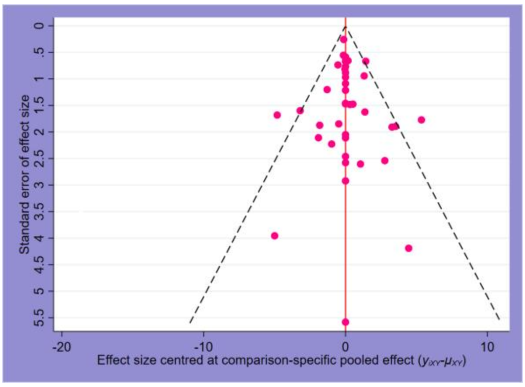

Figure S19 Funnel plot of WOMAC function (short-term)

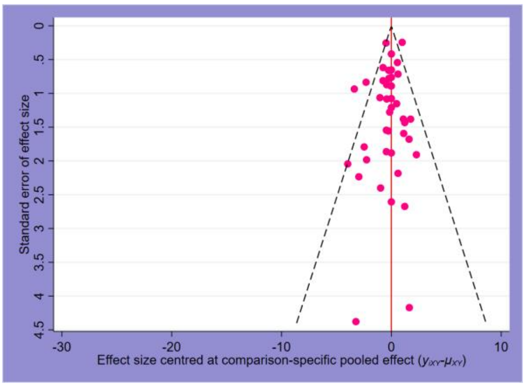

Figure S20 Funnel plot of WOMAC function (long-term)

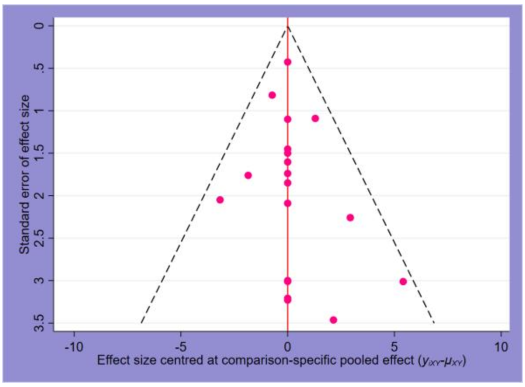

Figure S21 Funnel plot of ROM (short-term)

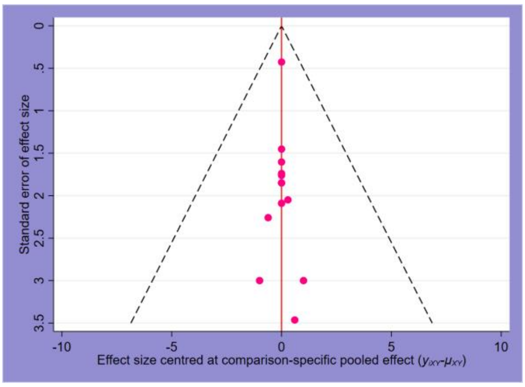

Figure S22 Funnel plot of ROM (long-term)

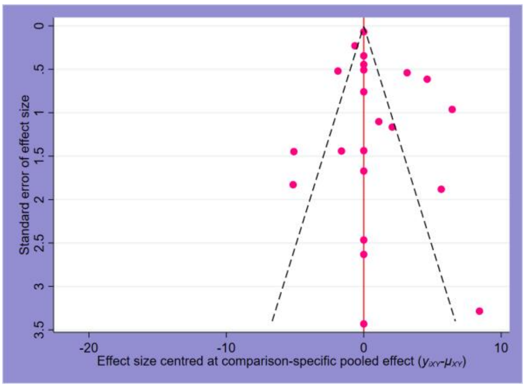

Figure S23 Funnel plot of IL-1β

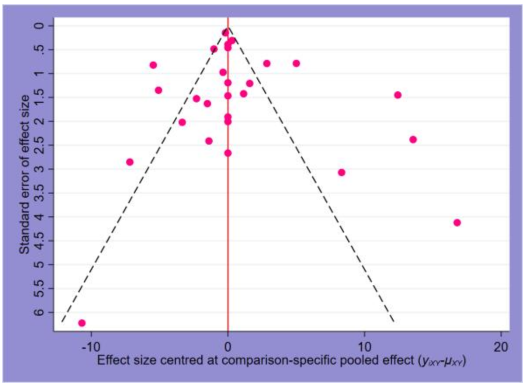

Figure S24 Funnel plot of TNF-α

The results of sensitivity analysis

(i)Sensitivity analysis after exclusion of high-risk literature

Note: Since the 5 high-risk literatures deleted only mentioned VAS (short-term), only this outcome indicator was analyzed.

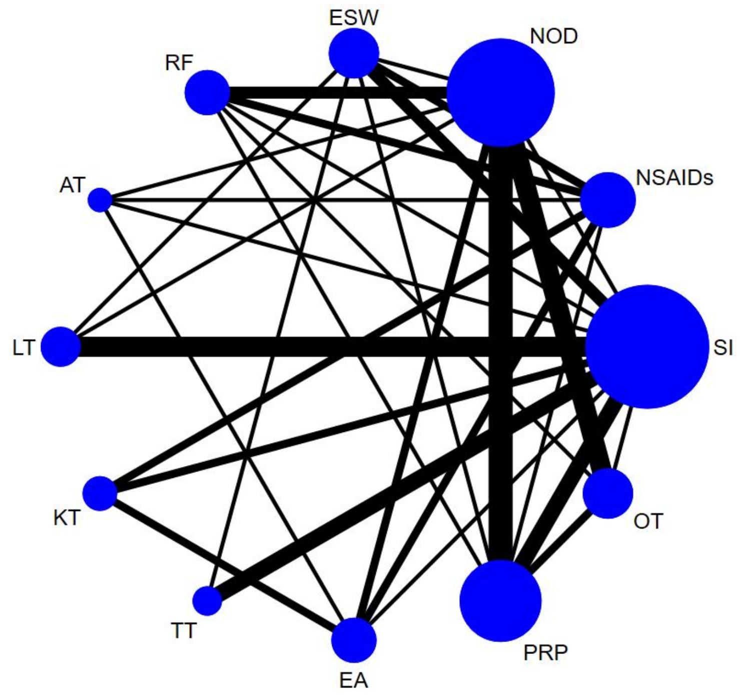

Figure S25 Evidence network diagram of VAS (short-term)

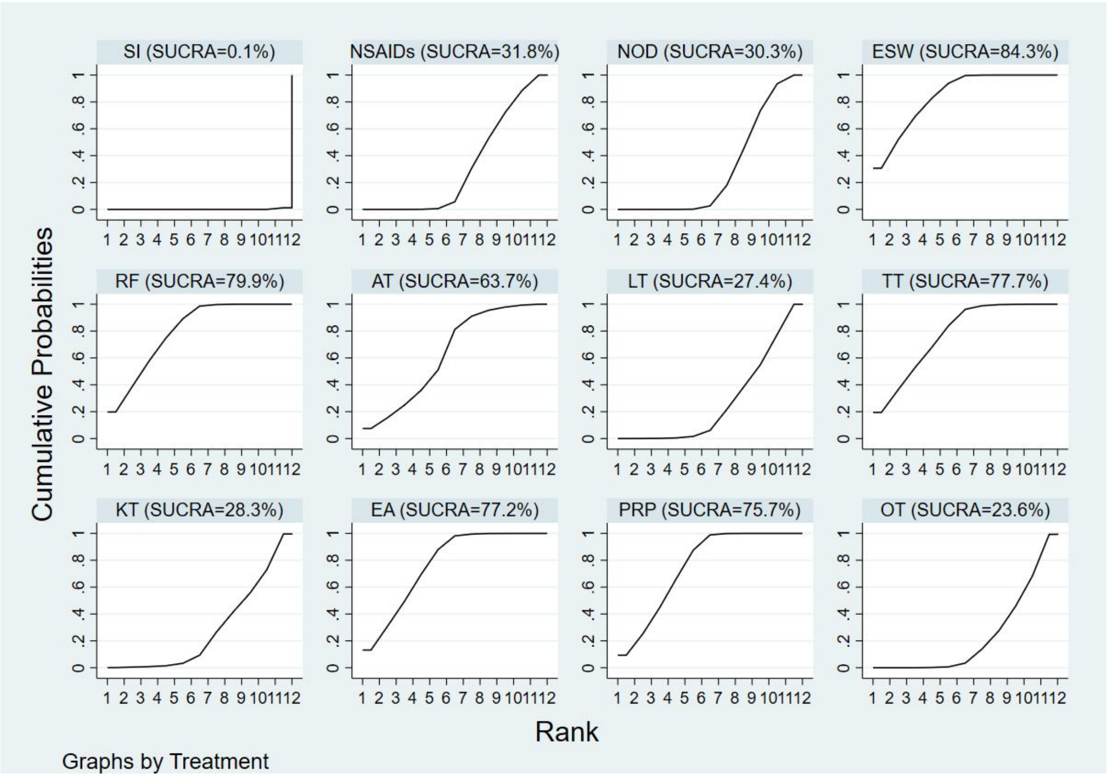

Figure S26 Ranking of SUCRA probabilities for short-term VAS

**ESW**											
-0.07<br>(-0.64,0.50)	**RF**										
-0.09<br>(-0.71,0.54)	-0.02<br>(-0.66,0.62)	**TT**									
-0.11<br>(-0.69,0.47)	-0.04<br>(-0.62,0.54)	-0.02<br>(-0.57,0.53)	**EA**								
-0.12<br>(-0.62,0.39)	-0.05<br>(-0.54,0.44)	-0.03<br>(-0.64,0.58)	-0.01<br>(-0.55,0.54)	**PRP**							
-0.29<br>(-1.00,0.42)	-0.23<br>(-0.94,0.48)	-0.21<br>(-0.95,0.54)	-0.19<br>(-0.84,0.47)	-0.18<br>(-0.86,0.50)	**AT**						
**-0.77<br>(-1.28,-0.26)**	**-0.70<br>(-1.21,-0.20)**	**-0.68<br>(-1.23,-0.14)**	**-0.66<br>(-1.16,-0.17)**	**-0.65<br>(-1.17,-0.14)**	-0.48<br>(-1.14,0.19)	**NSAIDs**					
**-0.80<br>(-1.29,-0.31)**	**-0.73<br>(-1.17,-0.30)**	**-0.71<br>(-1.30,-0.12)**	**-0.69<br>(-1.19,-0.20)**	**-0.69<br>(-1.06,-0.32)**	-0.51<br>(-1.14,0.13)	-0.03<br>(-0.52,0.46)	**NOD**				
**-0.85<br>(-1.52,-0.17)**	**-0.78<br>(-1.53,-0.03)**	-0.76<br>(-1.54,0.02)	-0.74<br>(-1.49,0.01)	**-0.73<br>(-1.43,-0.04)**	-0.55<br>(-1.40,0.30)	-0.08<br>(-0.81,0.66)	-0.05<br>(-0.74,0.65)	**KT**			
**-0.85<br>(-1.44,-0.27)**	**-0.79<br>(-1.43,-0.14)**	**-0.77<br>(-1.46,-0.08)**	**-0.75<br>(-1.40,-0.10)**	**-0.74<br>(-1.32,-0.16)**	-0.56<br>(-1.32,0.20)	-0.08<br>(-0.72,0.55)	-0.05<br>(-0.61,0.51)	-0.01<br>(-0.74,0.73)	**LT**		
**-0.91<br>(-1.53,-0.28)**	**-0.84<br>(-1.41,-0.28)**	**-0.82<br>(-1.53,-0.12)**	**-0.80<br>(-1.44,-0.16)**	**-0.79<br>(-1.31,-0.28)**	-0.61<br>(-1.37,0.14)	-0.14<br>(-0.76,0.48)	-0.11<br>(-0.56,0.34)	-0.06<br>(-0.85,0.73)	-0.05<br>(-0.73,0.62)	**OT**	
**-1.61<br>(-2.04,-1.17)**	**-1.54<br>(-2.04,-1.04)**	**-1.52<br>(-2.06,-0.98)**	**-1.50<br>(-2.00,-1.00)**	**-1.49<br>(-1.90,-1.08)**	**-1.31<br>(-1.95,-0.68)**	**-0.84<br>(-1.32,-0.36)**	**-0.81<br>(-1.22,-0.40)**	**-0.76<br>(-1.34,-0.18)**	**-0.75<br>(-1.22,-0.29)**	**-0.70<br>(-1.25,-0.14)**	**SI**

Figure S27 Network meta-analysis of VAS in short-term [SMD(95% CI)]

(ii) Sensitivity analysis after exclusion of small sample sizes literature (<50 cases)

Note: Since the 11 small sample articles deleted did not involve WOMAC pain (long-term), this outcome indicator was not analyzed.

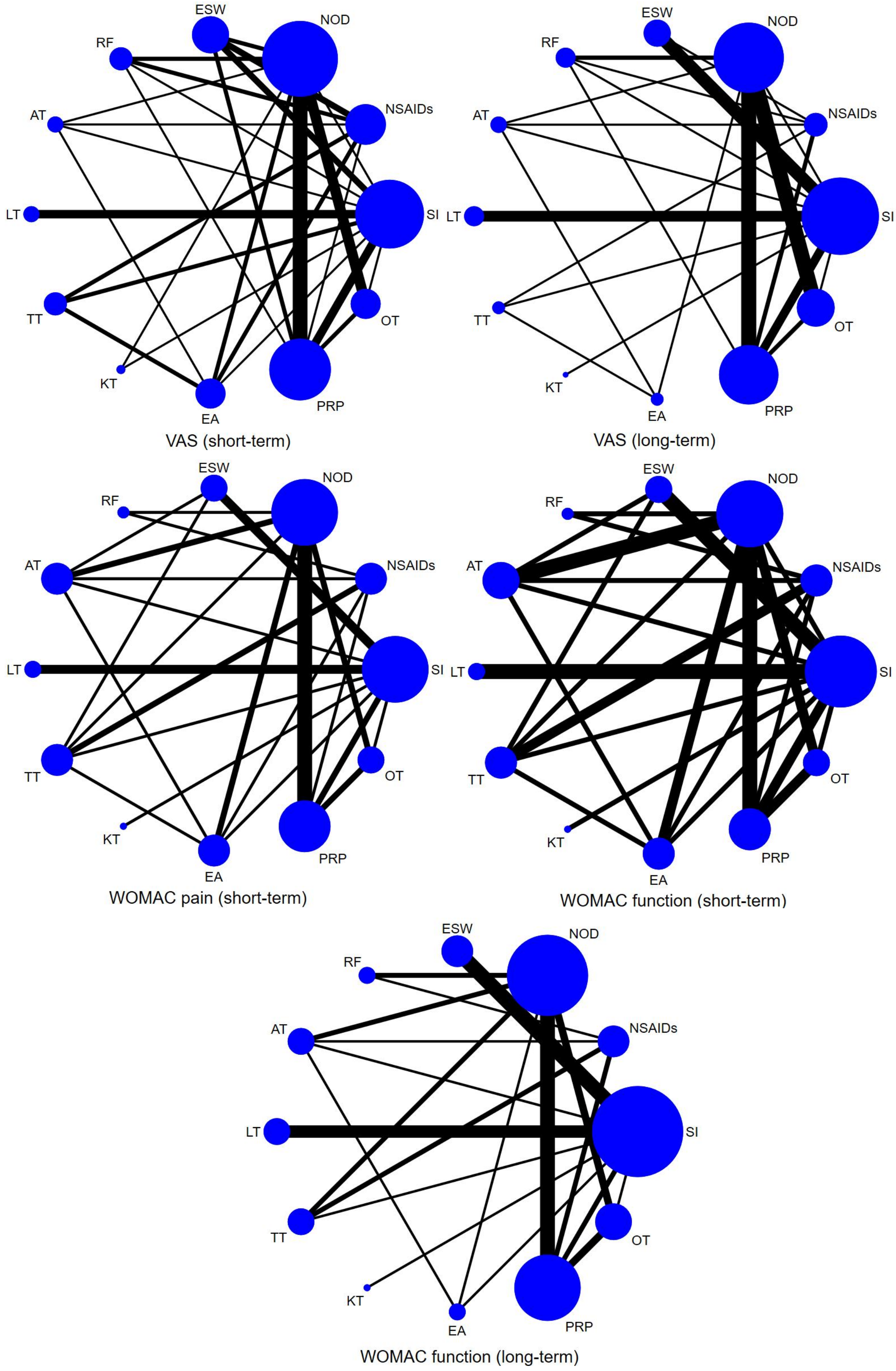

Figure S28 Evidence network diagram for each outcome indicator

Table S11 Ranking of SUCRA probabilities for each outcome indicator

Intervention	VAS (short-term)		VAS (long-term)		WOMAC Pain (short-term)		WOMAC Function (short-term)		WOMAC Function (long-term)	
	SUCRA	RANK	SUCRA	RANK	SUCRA	RANK	SUCRA	RANK	SUCRA	RANK
ESW	91.2	1	86.0	2	85.1	2	55.4	6	69.7	4
RF	84.2	2	88.4	1	97.5	1	65.8	5	86.1	2
AT	62.2	6	79.5	3	61.2	5	73.9	4	80.4	3
LT	29.1	9	37.5	8	68.5	4	76.6	3	42.8	8
TT	75.7	3	58.5	6	81.0	3	90.8	1	93.9	1
KT	18.8	11	43.0	7	27.6	10	40.6	8	46.1	7
EA	73.6	4	58.8	5	39.9	7	27.1	9	59.8	5
PRP	73.5	5	72.5	4	43.7	6	53.5	7	48.3	6
OT	21.0	10	30.0	9	36.2	8	43.0	8	33.2	9
NOD	32.5	8	25.6	10	25.6	11	25.4	10	24.0	10
NSAIDs	36.3	7	13.6	11	29.7	9	1.5	12	13.3	11
SI	2.0	12	5.9	12	4.1	12	10.3	11	2.4	12

VAS (Short-term)

VAS (Long-term)											
**ESW**	-0.05 (-0.79,0.69)	-0.52 (-1.41,0.37)	-0.51 (-1.44,0.42)	-0.25 (-0.86,0.36)	-0.12 (-0.93,0.69)	**-1.40 (-2.09,-0.71)**	**-1.15 (-1.80,-0.51)**	**-0.93 (-1.64,-0.23)**	**-1.08 (-1.81,-0.35)**	-0.84 (-2.29,0.62)	**-1.54 (-1.99,-1.08)**
-0.10 (-0.67,0.46)	**RF**	-0.57 (-1.48,0.34)	-0.56 (-1.49,0.36)	-0.30 (-0.87,0.27)	-0.17 (-0.99,0.64)	**-1.45 (-2.12,-0.78)**	**-1.20 (-1.76,-0.65)**	**-0.93 (-1.64,-0.23)**	**-1.13 (-1.80,-0.46)**	-0.89 (-2.40,0.63)	**-1.59 (-2.21,-0.97)**
-0.24 (-0.83,0.36)	-0.13 (-0.79,0.52)	**TT**	-0.01 (-0.87,0.85)	-0.27 (-1.10,0.55)	-0.40 (-1.32,0.52)	**-0.88 (-1.68,-0.08)**	-0.63 (-1.46,0.20)	-0.41 (-1.37,0.55)	-0.56 (-1.46,0.35)	-0.31 (-1.91,1.28)	**-1.01 (-1.81,-0.22)**
-0.27 (-0.81,0.27)	-0.17 (-0.76,0.43)	-0.03 (-0.56,0.50)	**EA**	-0.26 (-1.09,0.57)	-0.39 (-1.22,0.44)	**-0.89 (-1.77,-0.01)**	-0.64 (-1.45,0.16)	-0.42 (-1.42,0.57)	-0.57 (-1.46,0.32)	-0.33 (-1.94,1.29)	**-1.03 (-1.86,-0.19)**
-0.26 (-0.72,0.20)	-0.15 (-0.66,0.35)	-0.02 (-0.61,0.57)	0.01 (-0.51,0.53)	**PRP**	-0.13 (-0.83,0.58)	**-1.15 (-1.73,-0.58)**	**-0.91 (-1.27,-0.54)**	-0.69 (-1.39,0.01)	**-0.83 (-1.33,-0.34)**	-0.59 (-2.04,0.86)	**-1.29 (-1.73,-0.84)**
-0.46 (-1.13,0.22)	-0.35 (-1.07,0.37)	-0.22 (-0.94,0.51)	-0.19 (-0.82,0.45)	-0.20 (-0.85,0.45)	**AT**	**-1.28 (-2.03,-0.53)**	**-1.03 (-1.72,-0.34)**	-0.81 (-1.69,0.07)	**-0.96 (-1.74,-0.17)**	-0.71 (-2.26,0.83)	**-1.41 (-2.11,-0.71)**
**-0.94 (-1.38,-0.49)**	**-0.83 (-1.34,-0.33)**	**-0.70 (-1.23,-0.17)**	**-0.66 (-1.14,-0.19)**	**-0.68 (-1.16,-0.20)**	-0.48 (-1.12,0.16)	**NSAIDs**	-0.25 (-0.86,0.37)	-0.47 (-1.28,0.35)	-0.32 (-1.03,0.38)	-0.57 (-2.08,0.94)	-0.13 (-0.74,0.48)
**-1.01 (-1.46,-0.57)**	**-0.91 (-1.39,-0.43)**	**-0.78 (-1.35,-0.20)**	**-0.74 (-1.22,-0.26)**	**-0.76 (-1.09,-0.42)**	-0.56 (-1.18,0.06)	-0.08 (-0.54,0.38)	**NOD**	-0.22 (-0.95,0.51)	-0.07 (-0.49,0.34)	-0.32 (-1.78,1.15)	-0.38 (-0.87,0.11)
**-1.08 (-1.80,-0.36)**	**-0.97 (-1.75,-0.19)**	**-0.84 (-1.62,-0.06)**	**-0.81 (-1.56,-0.05)**	**-0.82 (-1.52,-0.12)**	-0.62 (-1.46,0.22)	-0.14 (-0.88,0.60)	-0.06 (-0.77,0.64)	**LT**	-0.14 (-0.95,0.66)	-0.10 (-1.58,1.38)	**-0.60 (-1.14,-0.06)**
**-1.23 (-1.84,-0.62)**	**-1.12 (-1.77,-0.48)**	**-0.99 (-1.70,-0.28)**	**-0.95 (-1.60,-0.31)**	**-0.97 (-1.49,-0.45)**	**-0.77 (-1.52,-0.02)**	-0.29 (-0.92,0.33)	-0.21 (-0.67,0.25)	-0.15 (-0.95,0.66)	**OT**	-0.24 (-1.75,1.26)	-0.46 (-1.05,0.14)
**-1.31 (-2.18,-0.44)**	**-1.21 (-2.11,-0.30)**	**-1.07 (-2.00,-0.14)**	**-1.04 (-1.93,-0.15)**	**-1.05 (-1.89,-0.22)**	-0.85 (-1.82,0.12)	-0.38 (-1.26,0.50)	-0.30 (-1.09,0.50)	-0.23 (-1.23,0.76)	-0.08 (-0.99,0.82)	**KT**	-0.70 (-2.08,0.68)
**-1.73 (-2.16,-1.30)**	**-1.63 (-2.16,-1.10)**	**-1.49 (-2.01,-0.97)**	**-1.46 (-1.94,-0.98)**	**-1.47 (-1.87,-1.08)**	**-1.27 (-1.89,-0.66)**	**-0.80 (-1.26,-0.33)**	**-0.72 (-1.12,-0.32)**	**-0.65 (-1.23,-0.08)**	-0.51 (-1.07,0.06)	-0.42 (-1.23,0.39)	**SI**

Figure S29 Network meta-analysis of VAS in short- and long-term [SMD(95% CI)]

**RF**											
-1.85 (-5.12,1.41)	**ESW**										
-2.22 (-5.23,0.80)	-0.36 (-2.62,1.89)	**TT**									
-2.98 (-6.67,0.71)	-1.13 (-3.96,1.71)	-0.76 (-3.69,2.16)	**LT**								
**-3.53 (-6.51,-0.55)**	-1.68 (-3.85,0.49)	-1.32 (-3.50,0.87)	-0.55 (-3.44,2.33)	**AT**							
**-4.44 (-7.30,-1.59)**	**-2.59 (-4.89,-0.29)**	**-2.23 (-4.36,-0.09)**	-1.46 (-4.25,1.32)	-0.91 (-2.93,1.11)	**PRP**						
**-4.60 (-7.62,-1.59)**	**-2.75 (-5.15,-0.35)**	**-2.39 (-4.44,-0.33)**	-1.62 (-4.56,1.31)	-1.07 (-3.12,0.98)	-0.16 (-2.26,1.93)	**EA**					
**-4.79 (-8.07,-1.51)**	**-2.93 (-5.66,-0.20)**	-2.57 (-5.21,0.07)	-1.81 (-4.93,1.32)	-1.26 (-3.79,1.28)	-0.35 (-2.43,1.74)	-0.18 (-2.79,2.42)	**OT**				
**-5.06 (-7.72,-2.41)**	**-3.21 (-5.68,-0.74)**	**-2.85 (-4.84,-0.86)**	-2.09 (-5.12,0.95)	-1.53 (-3.62,0.56)	-0.62 (-2.67,1.43)	-0.46 (-2.56,1.64)	-0.28 (-2.92,2.36)	**NSAIDs**			
**-5.50 (-10.25,-0.75)**	-3.65 (-7.77,0.47)	-3.29 (-7.47,0.90)	-2.52 (-6.82,1.77)	-1.97 (-6.12,2.18)	-1.06 (-5.15,3.03)	-0.90 (-5.09,3.29)	-0.72 (-5.04,3.61)	-0.44 (-4.70,3.83)	**KT**		
**-5.18 (-7.83,-2.53)**	**-3.33 (-5.59,-1.07)**	**-2.96 (-4.98,-0.95)**	-2.20 (-5.01,0.61)	-1.65 (-3.46,0.16)	-0.74 (-2.14,0.66)	-0.58 (-2.48,1.33)	-0.39 (-2.49,1.70)	-0.12 (-2.10,1.87)	0.32 (-3.79,4.43)	**NOD**	
**-6.70 (-9.68,-3.72)**	**-4.85 (-6.66,-3.04)**	**-4.49 (-6.44,-2.53)**	**-3.72 (-5.90,-1.54)**	**-3.17 (-5.06,-1.28)**	**-2.26 (-4.00,-0.52)**	**-2.10 (-4.07,-0.13)**	-1.92 (-4.16,0.33)	-1.64 (-3.76,0.48)	-1.20 (-4.90,2.50)	-1.52 (-3.30,0.26)	**SI**

Figure S30 Network meta-analysis of WOMAC pain in short-term [SMD(95% CI)]

	WOMAC function (Long-term)											
WOMAC function (Short-term)	**TT**	**-8.50 (-13.81,-3.18)**	**-7.33 (-13.11,-1.56)**	-2.57 (-7.75,2.61)	-1.40 (-7.08,4.27)	-4.13 (-9.77,1.50)	**-6.85 (-11.30,-2.41)**	-7.02 (-16.45,2.41)	-5.20 (-11.19,0.79)	**-9.52 (-13.73,-5.31)**	**-13.42 (-18.12,-8.72)**	**-11.24 (-15.61,-6.87)**
	-1.59 (-7.34,4.16)	**OT**	-1.17 (-6.68,4.35)	**-5.93 (-11.00,-0.85)**	**-7.09 (-12.71,-1.48)**	-4.36 (-9.72,0.99)	-1.65 (-5.37,2.07)	-1.48 (-10.75,7.79)	-3.30 (-9.12,2.52)	-1.02 (-4.82,2.78)	**-4.92 (-9.28,-0.56)**	-2.74 (-7.62,2.14)
	-1.94 (-8.23,4.35)	-0.35 (-7.24,6.54)	**LT**	-4.76 (-10.22,0.70)	-5.93 (-12.39,0.53)	-3.20 (-7.79,1.40)	-0.48 (-5.40,4.44)	-0.31 (-9.16,8.54)	-2.13 (-8.07,3.81)	-2.19 (-7.25,2.88)	**-6.09 (-9.46,-2.71)**	-3.90 (-9.55,1.74)
	-2.38 (-6.98,2.23)	-0.78 (-6.21,4.64)	-0.44 (-6.56,5.68)	**AT**	-1.17 (-6.75,4.42)	-1.56 (-6.86,3.74)	**-4.28 (-8.53,-0.04)**	-4.45 (-13.68,4.78)	-2.63 (-7.56,2.30)	**-6.95 (-10.92,-2.97)**	**-10.85 (-15.14,-6.56)**	**-8.67 (-13.14,-4.19)**
	-3.35 (-9.79,3.09)	-1.76 (-8.83,5.32)	-1.41 (-9.33,6.52)	-0.97 (-7.25,5.31)	**RF**	-2.73 (-9.05,3.59)	**-5.45 (-10.26,-0.63)**	-5.62 (-15.47,4.24)	-3.80 (-10.22,2.62)	**-8.11 (-12.46,-3.77)**	**-12.02 (-17.52,-6.52)**	**-9.83 (-14.65,-5.01)**
	-4.59 (-9.45,0.27)	-3.00 (-8.96,2.97)	-2.65 (-8.68,3.39)	-2.21 (-6.76,2.34)	-1.24 (-8.20,5.72)	**ESW**	-2.72 (-7.46,2.03)	-2.89 (-11.64,5.87)	-1.07 (-6.86,4.73)	**-5.38 (-10.27,-0.50)**	**-9.29 (-12.40,-6.17)**	**-7.10 (-12.59,-1.62)**
	**-4.70 (-9.37,-0.03)**	-3.11 (-7.59,1.37)	-2.76 (-8.80,3.27)	-2.33 (-6.66,2.00)	-1.36 (-7.59,4.88)	-0.11 (-5.07,4.84)	**PRP**	-0.17 (-9.10,8.76)	-1.65 (-6.82,3.52)	**-2.67 (-5.29,-0.04)**	**-6.57 (-10.15,-2.99)**	**-4.38 (-8.10,-0.67)**
	-7.15 (-16.33,2.02)	-5.56 (-15.15,4.03)	-5.21 (-14.62,4.19)	-4.78 (-13.82,4.27)	-3.80 (-14.15,6.54)	-2.56 (-11.56,6.43)	-2.45 (-11.44,6.54)	**KT**	-1.82 (-11.35,7.71)	-2.50 (-11.50,6.51)	-6.40 (-14.58,1.78)	-4.22 (-13.56,5.13)
	**-8.93 (-13.56,-4.29)**	**-7.34 (-13.07,-1.60)**	**-6.99 (-13.35,-0.63)**	**-6.55 (-10.96,-2.14)**	-5.58 (-12.17,1.01)	-4.34 (-9.56,0.88)	-4.23 (-8.94,0.49)	-1.78 (-11.00,7.44)	**EA**	-4.32 (-9.28,0.65)	**-8.22 (-13.11,-3.33)**	**-6.04 (-11.72,-0.35)**
	**-9.11 (-13.31,-4.91)**	**-7.52 (-12.08,-2.95)**	**-7.17 (-13.16,-1.18)**	**-6.73 (-10.33,-3.14)**	**-5.76 (-11.44,-0.09)**	-4.52 (-9.24,0.19)	**-4.41 (-7.78,-1.04)**	-1.96 (-10.92,7.00)	-0.18 (-4.22,3.85)	**NOD**	**-3.90 (-7.67,-0.14)**	-1.72 (-5.51,2.07)
	**-12.15 (-16.36,-7.95)**	**-10.56 (-15.60,-5.52)**	**-10.21 (-14.90,-5.52)**	**-9.78 (-13.69,-5.86)**	**-8.80 (-15.18,-2.43)**	**-7.56 (-11.36,-3.77)**	**-7.45 (-11.24,-3.66)**	-5.00 (-13.15,3.15)	-3.22 (-7.53,1.08)	-3.04 (-6.76,0.67)	**SI**	-2.18 (-6.70,2.33)
	**-15.27 (-19.70,-10.83)**	**-13.67 (-19.47,-7.88)**	**-13.33 (-19.98,-6.68)**	**-12.89 (-17.39,-8.39)**	**-11.92 (-17.59,-6.25)**	**-10.68 (-16.08,-5.28)**	**-10.56 (-15.12,-6.00)**	-8.11 (-17.51,1.28)	**-6.34 (-11.34,-1.34)**	**-6.16 (-10.49,-1.82)**	-3.11 (-7.79,1.56)	**NSAIDs**

Figure S31 Network meta-analysis of WOMAC function in short- and long-term [SMD(95% CI)]

The results of subgroup analysis

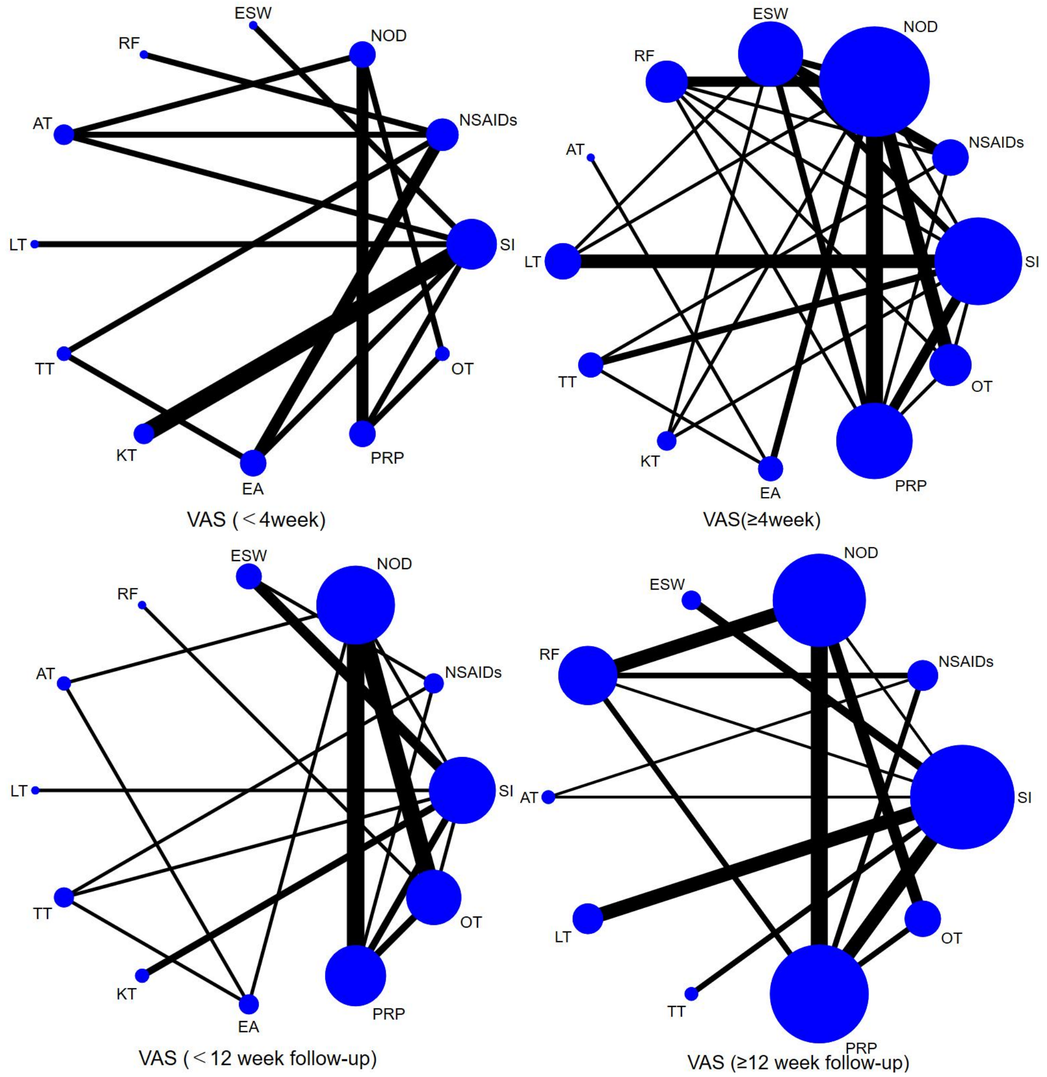

Figure S32 Evidence network diagram of VAS (subgroup analysis)

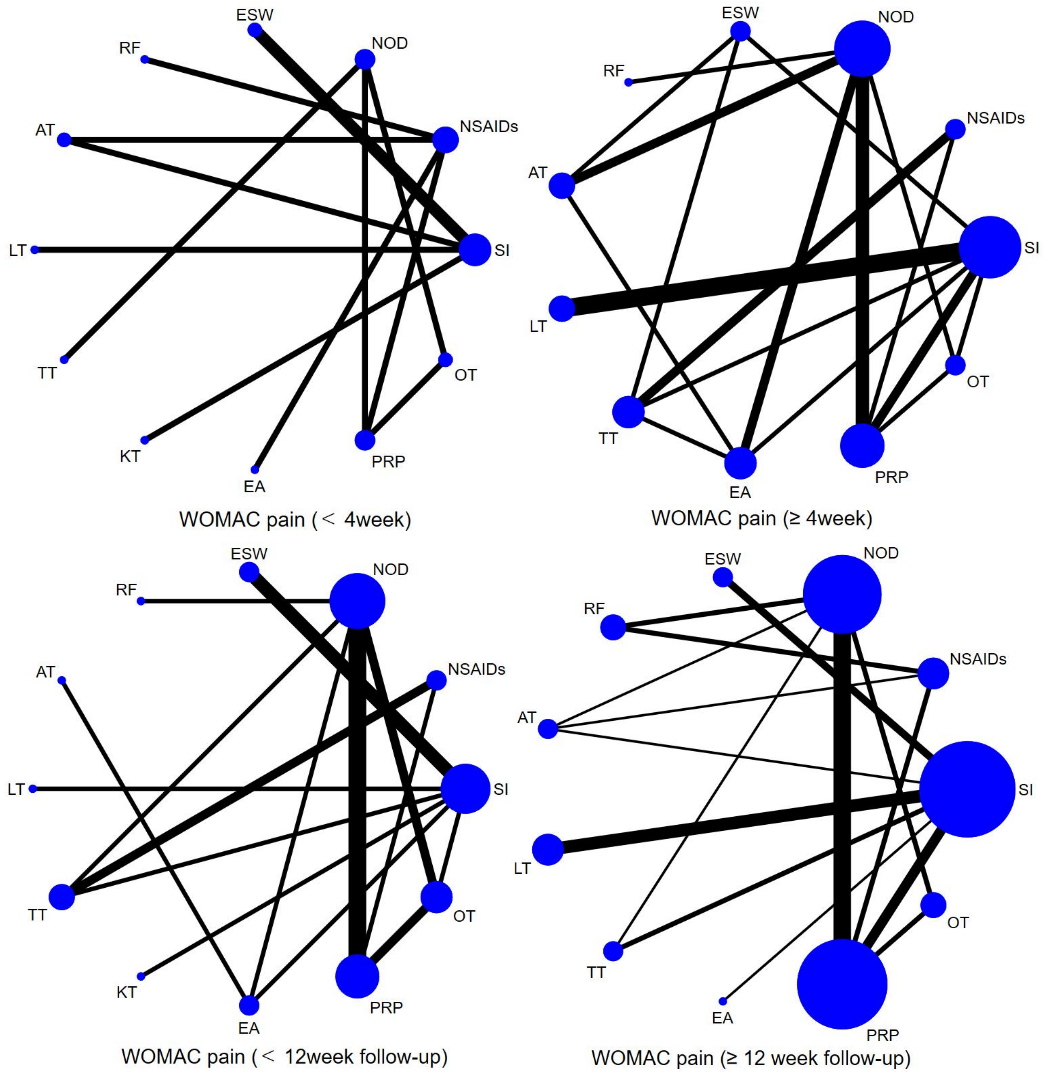

Figure S33 Evidence network diagram of WOMAC pain (subgroup analysis)

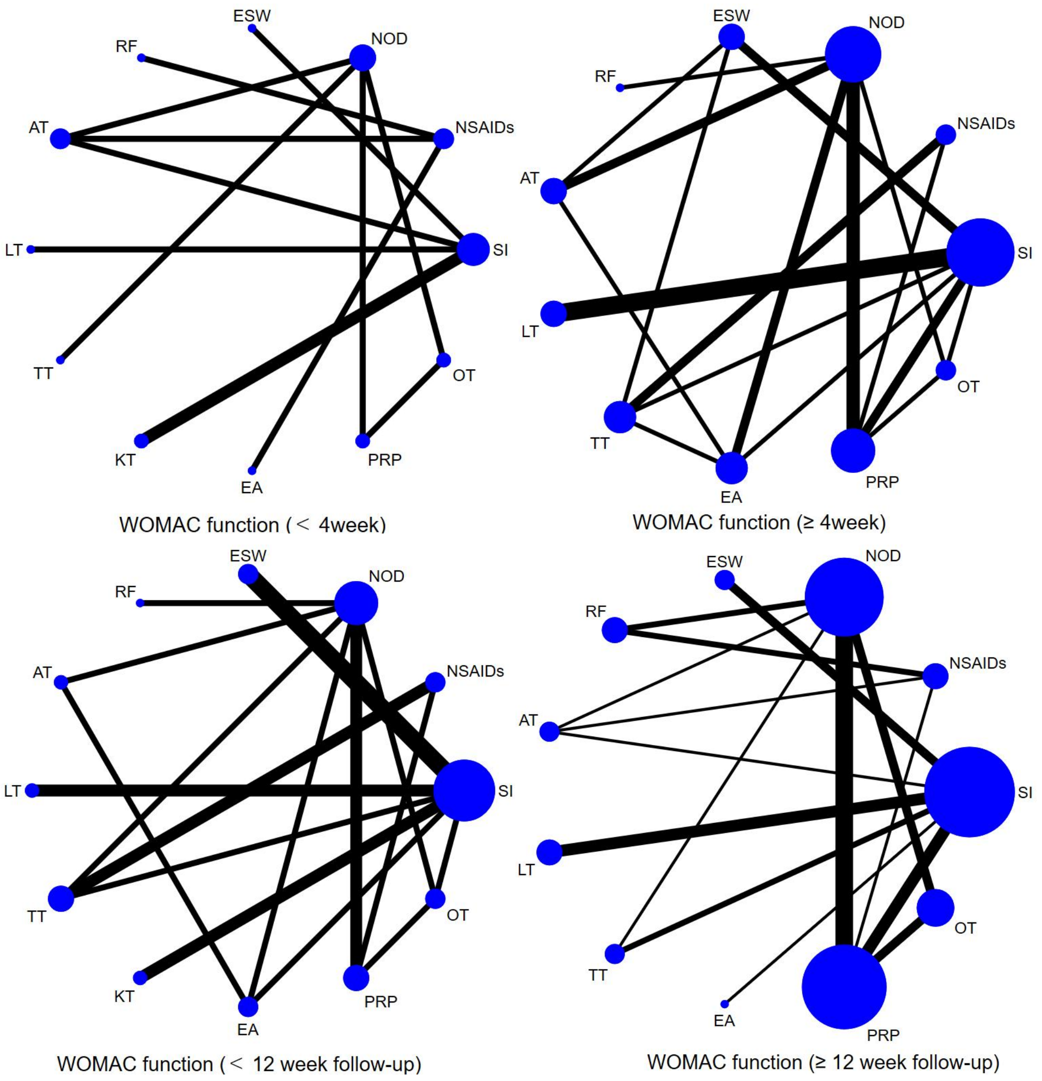

Figure S34 Evidence network diagram of WOMAC function (subgroup analysis)

Table S12 Ranking of SUCRA probabilities for each outcome indicator

Intervention	VAS (< 4week)		VAS (≥ 4week)		VAS follow-up (< 12week)		VAS follow-up (≥ 12week)		WOMAC pain (< 4week)		WOMAC pain (≥ 4week)	
	SUCRA	RANK	SUCRA	RANK	SUCRA	RANK	SUCRA	RANK	SUCRA	RANK	SUCRA	RANK
ESW	84.9	1	91.5	1	81.1	2	85.1	2	84.5	2	90.5	2
RF	82.0	2	84.7	2	82.3	1	90.0	1	89.2	1	92.8	1
AT	43.9	8	52.3	6	67.9	5	51.9	5	60.7	6	69.9	4
LT	48.9	6	27.4	9	35.4	8	44.0	7	67.9	5	47.8	6
TT	66.7	4	77.2	3	59.0	6	76.5	3	74.1	3	73.6	3
KT	37.7	9	31.2	8	33.8	9	-	-	3.0	12	-	-
EA	72.8	3	66.7	5	76.1	3	-	-	72.0	4	61.4	5
PRP	54.4	5	73.7	4	70.9	4	70.4	4	53.6	7	34.1	8
OT	23.3	11	49.3	7	44.6	7	45.2	6	27.0	9	39.1	7
NOD	32.3	10	22.4	11	31.5	10	23.5	8	41.3	8	29.6	9
NSAIDs	48.3	7	23.5	10	13.8	11	4.9	10	13.9	10	6.5	10
SI	4.9	12	0.1	12	3.4	12	8.5	9	12.8	11	4.8	11

Intervention	WOMAC pain (< 12week)		WOMAC pain (≥ 12week)		WOMAC function (< 4week)		WOMAC function (≥ 4week)		WOMAC function (< 12week)		WOMAC function (≥ 12week)	
	SUCRA	RANK	SUCRA	RANK	SUCRA	RANK	SUCRA	RANK	SUCRA	RANK	SUCRA	RANK
ESW	71.5	3	72.0	3	61.3	5	58.4	6	62.4	5	56.8	4
RF	97.2	1	89.1	1	58.5	6	64.1	5	89.9	2	90.8	2
AT	74.7	2	76.1	2	71.1	3	74.0	3	83.8	3	81.9	3
LT	63.0	4	66.0	4	66.0	4	65.5	4	45.3	7	48.9	6
TT	59.3	5	60.6	5	94.7	1	87.6	1	93.8	1	91.5	1
KT	40.8	9	-	-	31.7	9	-	-	30.4	9	-	-
EA	54.2	7	28.1	8	55.3	7	30.3	8	63.7	4	55.2	5
PRP	41.8	8	60.3	6	49.6	8	56.1	7	51.8	6	46.8	7
OT	55.1	6	39.2	7	71.5	2	74.2	2	35.2	8	43.7	8
NOD	20.8	10	25.6	10	29.6	10	27.1	9	25.7	10	23.6	9
NSAIDs	12.4	11	27.0	9	1.0	12	2.6	11	2.2	12	1.8	11
SI	9.3	12	6.1	11	9.7	11	10.2	10	15.6	11	9.0	10
